# High-resolution alignment of single-cell and spatial transcriptomes with CytoSPACE

**DOI:** 10.1038/s41587-023-01697-9

**Published:** 2023-03-06

**Authors:** Milad R. Vahid, Erin L. Brown, Chloé B. Steen, Wubing Zhang, Hyun Soo Jeon, Minji Kang, Andrew J. Gentles, Aaron M. Newman

**Affiliations:** 1 Institute for Stem Cell Biology and Regenerative Medicine, Stanford University, Stanford, CA USA; 2 Department of Biomedical Data Science, Stanford University, Stanford, CA USA; 3 Department of Cancer Immunology, Institute for Cancer Research, Oslo University Hospital, Oslo, Norway; 4 Department of Medical Genetics, Oslo University Hospital, Oslo, Norway; 5 Department of Computer Science, Stanford University, Stanford, CA USA; 6 Stanford Cancer Institute, Stanford University, Stanford, CA USA; 7 Department of Pathology, Stanford University, Stanford, CA USA; 8 Department of Medicine, Stanford Center for Biomedical Informatics Research, Stanford University, Stanford, CA USA

**Keywords:** Data integration, Software, Transcriptomics, Cancer microenvironment

## Abstract

Recent studies have emphasized the importance of single-cell spatial biology, yet available assays for spatial transcriptomics have limited gene recovery or low spatial resolution. Here we introduce CytoSPACE, an optimization method for mapping individual cells from a single-cell RNA sequencing atlas to spatial expression profiles. Across diverse platforms and tissue types, we show that CytoSPACE outperforms previous methods with respect to noise tolerance and accuracy, enabling tissue cartography at single-cell resolution.

## Abstract (web summary)

CytoSPACE maps individual cells from a reference single-cell RNA sequencing atlas to spatial transcriptomics data.

## Main

Single-cell spatial organization is a key determinant of cell state and function. For example, in human tumors, local signaling networks differentially impact individual cells and their surrounding microenvironments, with implications for tumor growth, progression and response to therapy^1–6^. Although spatial transcriptomics (ST) has become a powerful tool for delineating spatial gene expression in primary tissue specimens, commonly used platforms, such as 10x Visium, remain limited to bulk gene expression measurements, where each spatially resolved expression profile is derived from as many as ten cells or more^7^.

**Extended Data Fig. 1:**
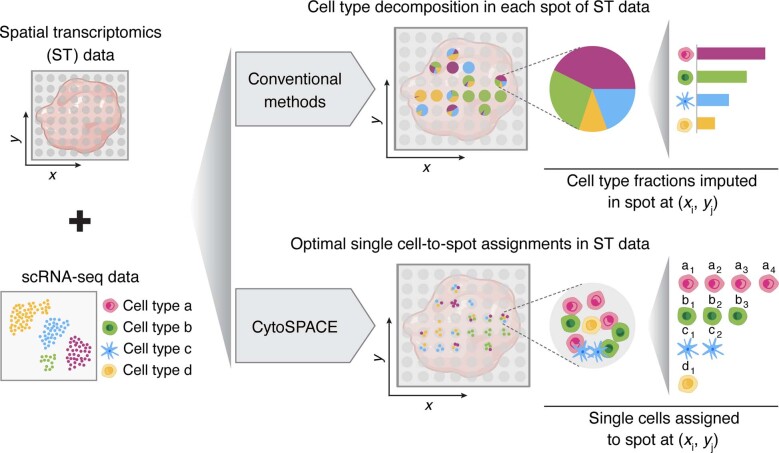
CytoSPACE versus conventional methods for decoding the cellular composition of bulk ST data. Most methods for deconvolving bulk ST data estimate cell type fractions using single-cell reference profiles (top). In contrast, CytoSPACE efficiently assigns individual single-cell transcriptomes to ST coordinates (that is, spots) using global optimization to minimize a correlation-based cost function. This enables downstream analysis of cell type proportions and single-cell transcriptional heterogeneity in spatial dimensions (bottom). The labels $a_1,...,d_1$ denote individual single cells of cell type $a,...,d$, respectively, assigned to the featured spot.

Accordingly, several computational methods have been developed to infer cellular composition in a given bulk ST sample^8–23^. Most such methods use reference profiles derived from single-cell RNA sequencing (scRNA-seq) data to deconvolve ST spots into a matrix of cell type proportions. However, these methods lack single-cell resolution, hindering the discovery of spatially defined cell states, their interaction patterns and their surrounding communities (Extended Data Fig. 1).

**Fig. 1:**
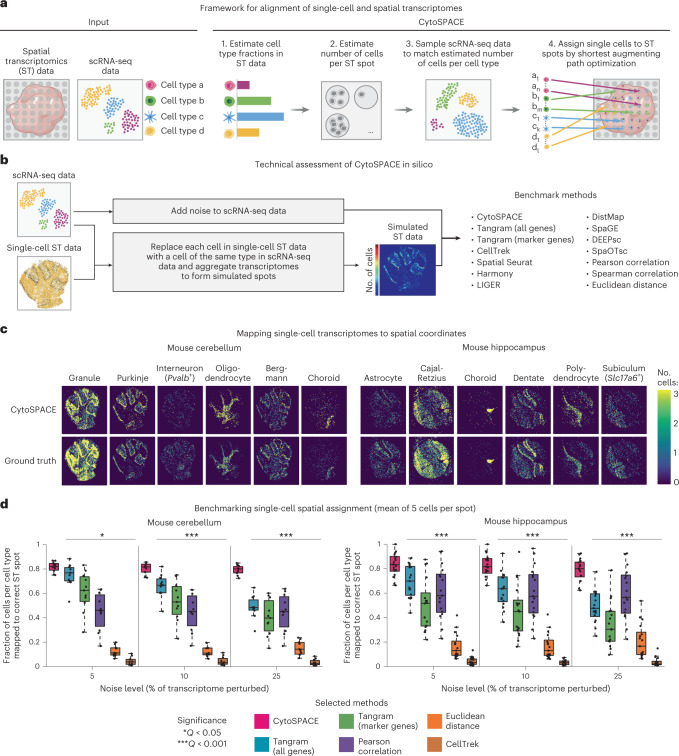
Development and technical assessment of CytoSPACE. **a**, Schematic of a typical CytoSPACE workflow. Given an ST dataset *A* and an annotated scRNA-seq dataset *B*, where the latter covers major cell types in *A*, CytoSPACE consists of the following key steps: (1) application of an existing ST deconvolution method (for example, Spatial Seurat or RCTD) to estimate cell type fractions in *A* using reference profiles from *B*; (2) estimation of the number of cells per spot in *A*; (3) sampling of *B* to match the inferred number of cells per cell type in *A*; and (4) alignment of single-cell and spatial transcriptomes (*B*→*A*) using shortest augmenting path-based optimization. The labels *a*_1_,*a*_*n*_,...,*d*_1_,*d*_*n*_ denote individual single cells of cell type *a*,*a*,…,*d,d*, respectively. **b–d**, Technical assessment of CytoSPACE. **b**, Framework for evaluating CytoSPACE using simulated ST datasets with fully defined single-cell composition and spot resolution (Methods). **c**, Heat maps depicting CytoSPACE performance for aligning scRNA-seq data (with 5% added noise) to spatial locations in ST datasets simulated with five cells per spot, on average (Methods). Only cell types with distinct spatial structure are shown here for clarity. **d**, Performance across distinct methods, mouse brain regions and noise levels for assigning individual cells to the correct spot in simulated ST datasets (Methods). Each point represents a single cell type (mouse cerebellum, *n* = 11; mouse hippocampus, *n* = 17). The box center lines, box bounds and whiskers indicate the medians, first and third quartiles and minimum and maximum values within 1.5× the interquartile range of the box limits, respectively. Statistical significance was assessed relative to CytoSPACE using a two-sided paired Wilcoxon test. The resulting *P* values were Benjamini–Hochberg adjusted for each noise level and tissue type combination and reported as the maximum *Q* value (*Q < 0.05 and ***Q < 0.001). Performance for all 13 evaluated methods is provided in Extended Data Fig. 4. Raw data are provided in Supplementary Table 2.

To address this challenge, we developed cellular (Cyto) Spatial Positioning Analysis via Constrained Expression alignment (CytoSPACE), an efficient computational approach for mapping individual cells from a reference scRNA-seq atlas to precise spatial locations in a bulk or single-cell ST dataset (Fig. 1a and Extended Data Fig. 1). Unlike related methods^24,25^, we formulate single-cell/spot assignment as a convex optimization problem and solve this problem using the Jonker–Volgenant shortest augmenting path algorithm^26^. Our approach guarantees an optimal mapping result while exhibiting improved noise tolerance (Methods). The output is a reconstructed tissue specimen with both high gene coverage and spatially resolved scRNA-seq data suitable for downstream analysis, including the discovery of context-dependent cell states. On both simulated and real ST datasets, we found that CytoSPACE substantially outperforms related methods for resolving single-cell spatial composition.

CytoSPACE proceeds in four main steps (Fig. 1a). First, to account for the disparity between scRNA-seq and ST data in the number of cells per cell type, two parameters are required: (1) the fractional abundance of each cell type within the ST sample and (2) the number of cells per spot. The former is determined using an external deconvolution tool, such as Spatial Seurat^14^, RCTD^18^, SPOTlight^20^, cell2location^27^ or CIBERSORTx^28^. By default, the latter is directly inferred by CytoSPACE using an approach for estimating RNA abundance, although alternative methods, including cell segmentation approaches^29,30^, can also be used (Methods). Once both parameters are estimated, the scRNA-seq dataset is randomly sampled to match the predicted number of cells per cell type in the ST dataset. Upsampling is done for cell types with insufficient representation, either by drawing with replacement or by introducing placeholder cells (Methods). Finally, CytoSPACE assigns each cell to spatial coordinates in a manner that minimizes a correlation-based cost function constrained by the inferred number of cells per spot via a shortest augmenting path optimization algorithm. An efficient integer programming approximation method that yields similar results is also provided^31^ (Methods).

**Extended Data Fig. 2:**
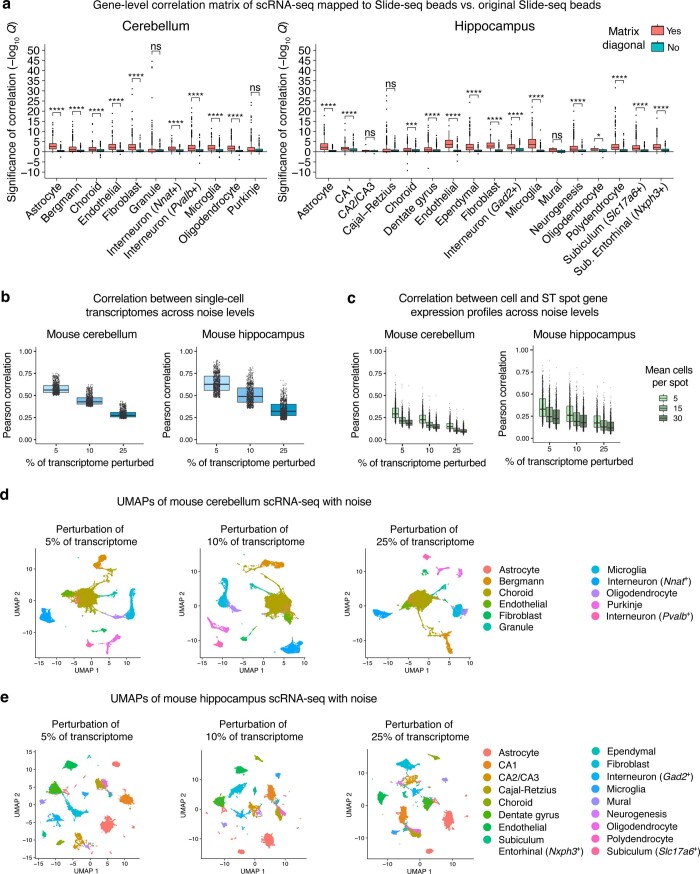
Maintenance of gene-level spatial dependencies in simulated ST data and impact of controlled noise on scRNA-seq query data. **a**, Pearson correlation analysis of log_2_ expression levels in (i) scRNA-seq mapped to Slide-seq beads (as part of simulated ST dataset construction, Methods) vs. (ii) the original Slide-seq beads. For each cell type, correlations were divided into two groups comparing: (i) correlations of the same gene across cells (matrix diagonal) with (ii) correlations of non-matching genes across cells (off-diagonal entries of the correlation matrix). Correlation p-values were corrected using the Benjamini-Hochberg method within each cell type and plotted as –log_10_ q-values, which were multiplied by –1 for negative correlations. Given practical considerations, we randomly selected 1,000 genes and a maximum of 1,000 cells per cell type. Group comparisons were evaluated using a one-sided Wilcoxon test relative to matching genes (matrix diagonal). The resulting p-values were Benjamini-Hochberg adjusted separately for each brain region and shown as q-values. *Q < 0.05; ***Q < 0.001; ****Q < 0.0001; ns, not significant. Sub., Subiculum. **b**, Box plots showing the effect of adding noise to the scRNA-seq query datasets used in simulation experiments. In brief, single-cell expression profiles of mouse cerebellum and hippocampus were perturbed by adding noise sampled from an exponentiated normal distribution to randomly selected genes, comprising 5% to 25% of each cell’s original transcriptome (x-axis, Methods). Concordance between the original and perturbed transcriptome in log_2_ space for 1,000 randomly sampled cells per scRNA-seq dataset (mouse cerebellum, left; mouse hippocampus, right), expressed as Pearson correlation coefficient (y-axis). **c**, Same as b but showing Pearson correlation (y-axis) between 1,000 randomly selected single-cell transcriptomes after the addition of noise (x-axis) and their corresponding ground truth ST spot transcriptomes, for different mean spot resolutions. Pearson correlation was determined in log_2_ space. **d-e**, UMAPs of scRNA-seq after the addition of noise for mouse cerebellum (d) and mouse hippocampus (e) datasets. Importantly, cell type clusters are maintained across the range of considered perturbations. The box center lines, box bounds, and whiskers in panels a–c indicate the medians, first and third quartiles and minimum and maximum values within 1.5× the interquartile range of the box limits, respectively.

To test the performance of CytoSPACE, we began by simulating ST datasets with fully defined single-cell composition. For this purpose, we leveraged previously published mouse cerebellum (*n* = 11 major cell types) and hippocampus (*n* = 17 major cell types) data generated using Slide-seq, a platform with high spatial resolution (approximately single-cell) but limited gene coverage^32^ (Fig. 1b and Supplementary Table 1). To increase transcriptome representation while maintaining spatial dependencies, we first replaced each Slide-seq bead with the most correlated single-cell expression profile of the same cell type derived from an scRNA-seq atlas of the same brain region^33^ (Extended Data Fig. 2a and Methods). We then superimposed a spatial grid with tunable dimensions to pool single-cell transcriptomes into pseudo-bulk transcriptomes. This was done across a range of realistic spot resolutions (mean of 5, 15 and 30 cells per spot). To guarantee a unique spatial address for every cell in the scRNA-seq query dataset, we created a paired scRNA-seq atlas from the cells underlying each pseudo-bulk ST array. Finally, to emulate technical and platform-specific variation between scRNA-seq and ST datasets, we added noise in varying amounts to the scRNA-seq data (Extended Data Fig. 2b–e and Methods). Collectively, these datasets allow rigorous assessment of cell-to-spot alignment, including orthogonal approaches for studying alignment quality (Supplementary Fig. 1).

**Extended Data Fig. 3:**
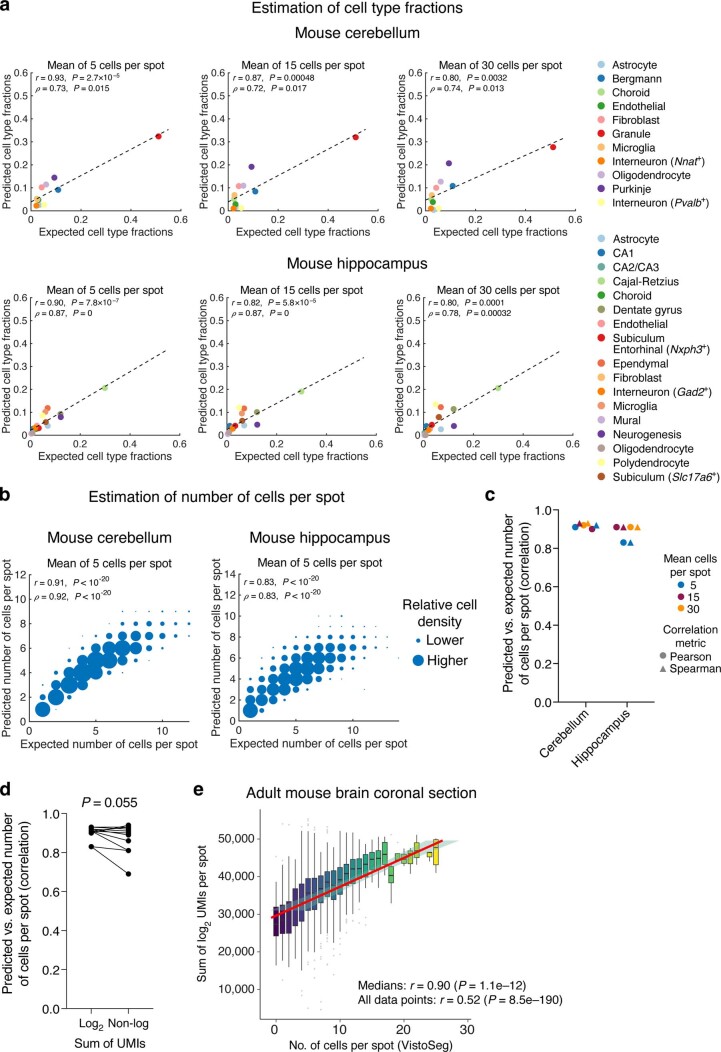
Estimation of cell type fractions and the number of cells per spot in bulk ST data. **a**, Application of Spatial Seurat to infer cell type fractions in simulated ST datasets (Methods). Scatter plots show ground truth cell type fractions (x-axis) versus estimated fractions (y-axis) for simulated ST data of mouse cerebellum (top) and hippocampus (bottom) sections with different spot resolutions. Single-cell RNA sequencing data were first perturbed with the addition of noise to 5% of the transcriptome, as described in Methods. **b**, Scatter plot showing the number of cells per spot estimated by CytoSPACE in simulated ST datasets (y-axis; Methods) versus ground truth (x-axis) at a mean of 5 cells per spot for mouse cerebellum and hippocampus sections. Relative density is depicted by point size. Concordance and significance were assessed by Pearson *r* or Spearman ρ and a two-sided *t* test, respectively. **c**, Same as b but showing correlation coefficients (Pearson and Spearman) for all analyzed spot resolutions. All correlations are significant (*P* < 10^–20^). **d**, Paired analysis showing the difference in performance between log_2_ adjustment and the non-log linear scale for predicting the number of cells per spot for all six combinations of spot resolutions in simulated ST datasets (mean of 5, 15, and 30) for Pearson and Spearman correlation coefficients. Statistical significance was calculated with a two-sided paired Wilcoxon test. **e**, Concordance between the number of cells per spot imputed by the default RNA-based approach implemented in CytoSPACE (y-axis) and a cell segmentation algorithm (VistoSeg) respectively applied to paired gene expression data and a histological image of an adult mouse brain coronal sample profiled by 10x Visium. The box center lines, box bounds, and whiskers indicate the medians, first and third quartiles and minimum and maximum values within 1.5× the interquartile range of the box limits, respectively. Linear regression, shown with a 95% confidence interval, was applied to the box plot medians. In panels a and b, concordance was assessed by Pearson correlation (*r*), Spearman correlation (*ρ*), and/or linear regression (dashed lines). A two-sided t-test was used to assess whether each correlation result was significantly nonzero. No adjustments for multiple comparisons were made.

Next, we evaluated methods for CytoSPACE parameter inference. For cell type enumeration, we employed Spatial Seurat, which showed strong concordance with known global proportions in simulated ST datasets (Extended Data Fig. 3a). To approximate the number of cells per spot, we implemented a simple approach based on RNA abundance estimation (Methods). This approach was correlated with ground truth expectations in simulated ST data and cell segmentation analysis^29^ of the matching histological image from real ST data (Extended Data Fig. 3b–e and Methods).

We then benchmarked CytoSPACE against 12 previous methods (Methods), including two recently described algorithms for scRNA-seq and ST alignment: Tangram, which integrates scRNA-seq and ST data via maximization of a spatial correlation function using non-convex optimization^24^; and CellTrek, which uses Spatial Seurat^14^ to identify a shared embedding between scRNA-seq and ST data and then applies random forest modeling to predict spatial coordinates^25^. We also assessed naive approaches, including Pearson correlation and Euclidean distance. To compare outputs, each cell was assigned to the spot with the highest score (all approaches but CellTrek) or the spot with the closest Euclidean distance to the cell’s predicted spatial location (CellTrek only). The full benchmarking analysis is provided in the supplement; further details are in Methods.

**Extended Data Fig. 4:**
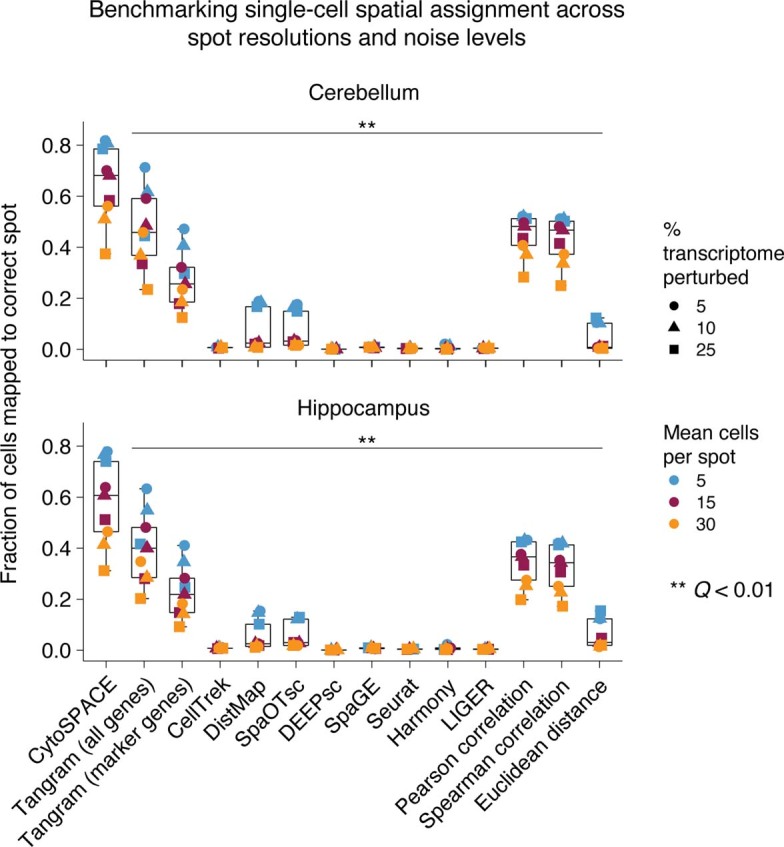
Extended benchmarking analysis on simulated ST data (related to Fig. 1d). Box plots depicting the fraction of all single-cell transcriptomes assigned to the correct ST spot, shown for different spot resolutions (mean of 5, 15, and 30 cells per spot) and scRNA-seq noise levels (perturbations added to 5%, 10%, and 25% of the transcriptome) for an extended array of 13 methods. Raw data are provided in Supplementary Table 2. Statistical significance was determined using a two-sided paired Wilcoxon test relative to CytoSPACE. P-values were corrected using the Benjamini-Hochberg method and are expressed as q-values (**Q < 0.01). The box center lines, box bounds, and whiskers indicate the medians, first and third quartiles and minimum and maximum values within 1.5× the interquartile range of the box limits, respectively.

Across multiple evaluated noise levels and cell types, CytoSPACE achieved substantially higher precision than other methods for mapping single cells to their known locations in simulated ST datasets (Fig. 1c,d, Extended Data Fig. 4, Supplementary Fig. 2 and Supplementary Table 2). This was true for multiple spatial resolutions independent of brain region, both for individual cell types and across all evaluable cells (Fig. 1d and Extended Data Fig. 4). We also obtained similar results with an independent method for determining cell type abundance in ST data (RCTD^18^) (Supplementary Fig. 3).

**Extended Data Fig. 5:**
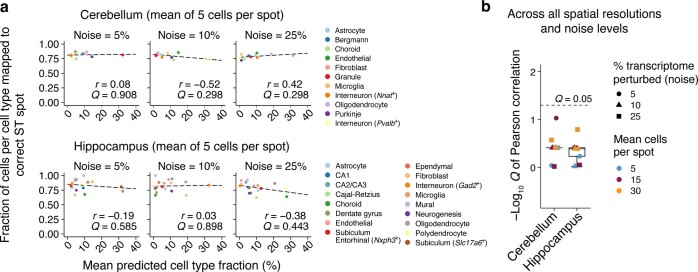
Association between CytoSPACE performance and inferred global cell type abundance in simulated ST datasets. **a**, Scatter plots comparing single-cell mapping accuracy in simulated ST datasets (with a mean of 5 cells per spot) with mean cell type fractional abundances inferred by Spatial Seurat for all cell types and noise levels (Methods). Linearity was determined by Pearson correlation. **b**, Same as the left panel but summarizing Pearson correlation significance values across all evaluated simulated ST datasets, spot resolutions, and noise levels. The box center lines, box bounds, and whiskers indicate the medians, first and third quartiles and minimum and maximum values within 1.5× the interquartile range of the box limits, respectively. In both panels, a two-sided t-test was used to assess whether each correlation coefficient was significantly nonzero. P-values were corrected using the Benjamini-Hochberg method and expressed as q-values.

**Extended Data Fig. 6:**
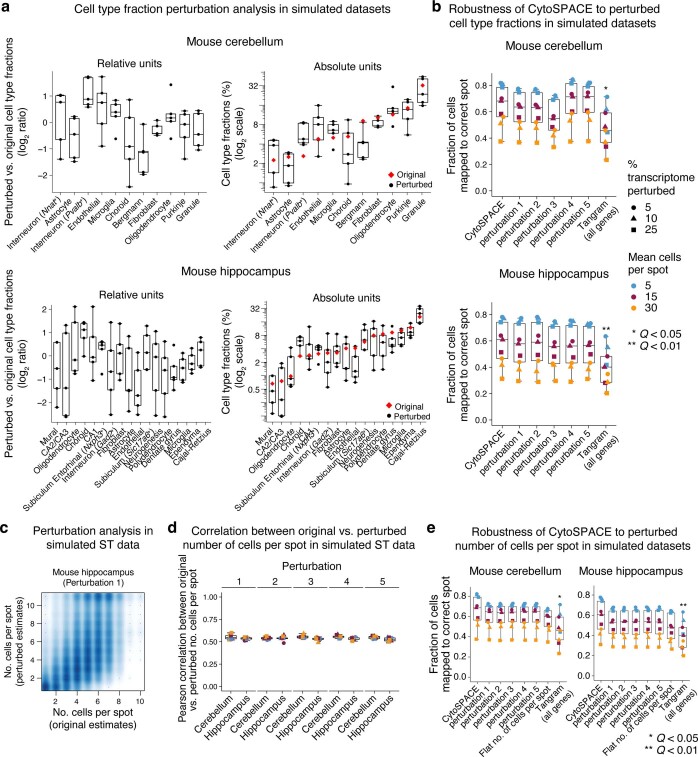
Impact of perturbing estimates of cell type fractional abundance and the number of cells per spot. **a–b**, Effect of perturbing fractional abundance estimates in simulated ST datasets. **a**, Box plots showing the effect of perturbation on cell type fractional abundance estimates over five separate trials, expressed relative to the original estimates (left) and in absolute units (right) for mouse cerebellum (top) and hippocampus (bottom) datasets with a mean of 5 cells per spot and 5% noise added to scRNA-seq query datasets (Methods). **b**, Box plots showing CytoSPACE performance on simulated ST datasets before and after perturbing cell type fractions for all spot resolutions and scRNA-seq noise levels (Methods). **c-e**, Effect of perturbing estimates of the number of cells per spot in simulated ST datasets. **c**, Scatter plot showing the effect of controlled perturbation on the estimated number of cells per spot for a representative simulated ST dataset (mouse hippocampus with a mean of 5 cells per spot; Methods). **d**, Box plots showing Pearson correlations between perturbed and original estimates of the number of cells per spot for all evaluated simulated ST datasets across five trials. **e**, Box plots showing CytoSPACE performance on all simulated ST datasets before and after perturbing estimates of the number of cells per spot (related to panel d). Point shapes and colors in d and e are defined in b. Group comparisons in panels b and e were performed using a two-sided paired Wilcoxon test for each CytoSPACE result versus each method in Extended Data Fig. 4, with ‘Tangram (all genes)’ shown as a representative example. P-values were corrected using the Benjamini-Hochberg method and are expressed as q-values (*Q < 0.05; **Q < 0.01). Q-values shown are inclusive of comparisons between CytoSPACE results and all benchmarked methods in Extended Data Fig. 4. The box center lines, box bounds, and whiskers in a, b, d, and e indicate the medians, first and third quartiles and minimum and maximum values within 1.5× the interquartile range of the box limits, respectively.

We next assessed the robustness of CytoSPACE to variation in key input parameters (steps 1–3 in Fig. 1a). First, we considered estimated cell type abundance, which ranged from a mean of 0.025% to 32% in simulated ST datasets (Extended Data Fig. 5). Despite this range, we observed no significant correlation with mapping precision (Extended Data Fig. 5). Next, we performed experiments in which estimates of (1) cell type abundance and (2) the number of cells per spot were systematically perturbed (Methods). In all cases, CytoSPACE continued to outperform previous methods (Extended Data Fig. 6). Lastly, we tested output stability when sampling the scRNA-seq query dataset with different seeds (step 3 in Fig. 1a) and when using different distance metrics to calculate the CytoSPACE cost function. Across multiple runs and distance metrics, results remained consistent (Supplementary Fig. 4). Collectively, these data highlight the robustness of CytoSPACE and underscore its potential to deliver improved spatial mapping of scRNA-seq data.

**Fig. 2:**
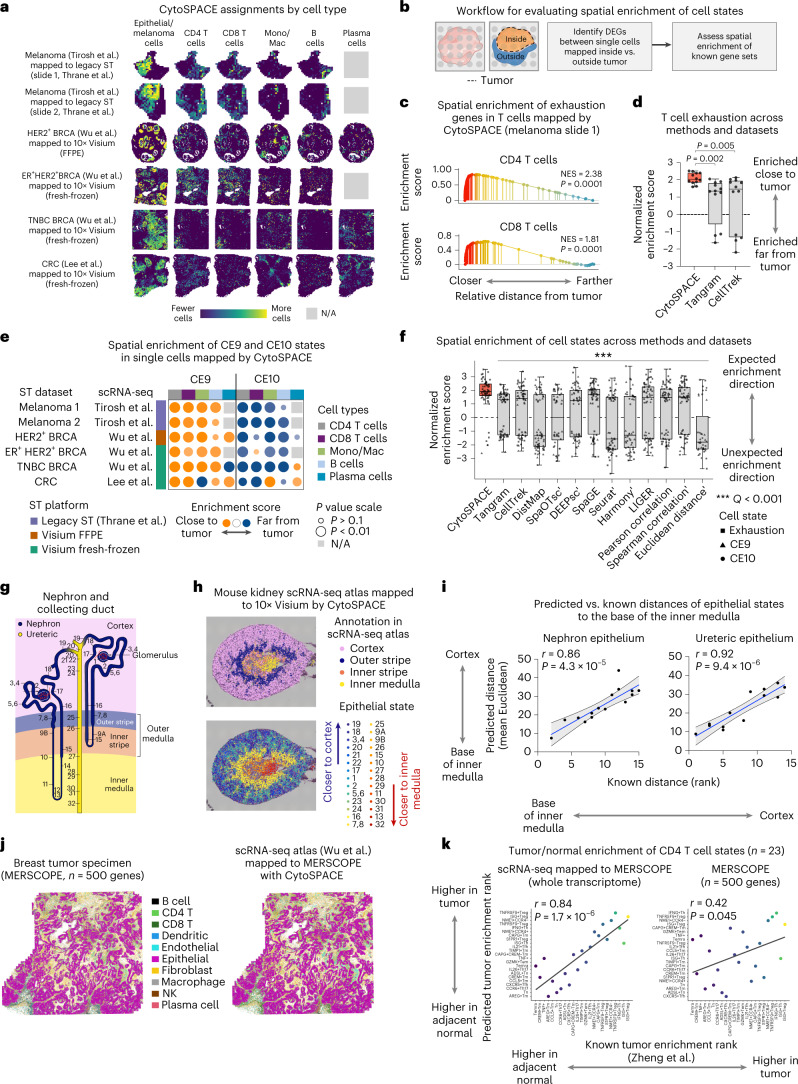
Single-cell cartography across diverse tissue types and platforms with CytoSPACE. **a**, scRNA-seq tumor atlases mapped onto clinically matched ST datasets by CytoSPACE (see also Supplementary Fig. 5). BRCA, breast cancer; CRC, colorectal cancer; N/A, missing from author-supplied annotations. **b**, Workflow for evaluating spatial enrichment in the tumor core or periphery. DEGs, differentially expressed genes. **c**, Spatial enrichment of T cell exhaustion genes in T cell transcriptomes mapped by CytoSPACE to a melanoma sample (row 1, **a**). NES, normalized enrichment score. **d**, Same as **c** but showing NES for six scRNA-seq/ST pairs (*n* = 12 values per box) and three methods. **e**, Spatial enrichments of CE9-specific and CE10-specific cell states in data mapped by CytoSPACE and analyzed by pre-ranked GSEA. Datasets without annotations are indicated in grey. **f**, Same as **d** and **e** but across 13 methods and 66 combinations of dataset pairs and cell states. To unify the expected enrichment direction of cell states, NES values for CE10 were multiplied by −1. Methods indicated by a prime symbol failed to map all evaluated cell types to regions both closer and farther from tumor cells, precluding the use of GSEA on the affected cell types. In such cases, paired Wilcoxon tests were performed relative to CytoSPACE but ignoring N/As. Underlying data are provided in Supplementary Table 7. **g**, Schematic of the mouse nephron and collecting duct system. Known locations of epithelial states are denoted by numbers (for phenotype labels, see Supplementary Table 8), recreated from https://cello.shinyapps.io/kidneycellexplorer/. **h**, Top: epithelial cell transcriptomes from a mouse kidney scRNA-seq atlas mapped onto a 10x Visium sample of normal mouse kidney by CytoSPACE, shown using jitter within assigned spots. Bottom: same as above but colored by known distance to the inner medulla (state 32; Supplementary Table 8). States 12 and 14 were imputed with zero abundance and not mapped. **i**, Concordance between predicted and known distances of each epithelial state to the base of the inner medulla. **j**, Left: MERSCOPE profile of a breast cancer specimen, colored by cell type. Right: scRNA-seq data^37^ mapped to the MERSCOPE profile by CytoSPACE, with previously annotated cell types from the scRNA-seq atlas distinguished by color. **k**, Enrichment of CD4 T cell states within tumor regions (pre-ranked GSEA), comparing scRNA-seq data mapped to MERSCOPE (CytoSPACE) with MERSCOPE alone (for underlying data, see Supplementary Table 9). Color scale is defined in Extended Data Fig. 10i. Two-sided nominal *P* values in **c** and **f** were determined by GSEA. In **d** and **f**, the box center lines, box bounds and whiskers denote the medians, first and third quartiles and minimum and maximum values within 1.5× the interquartile range of the box limits, respectively. Group comparisons in **d** and **f** were determined relative to CytoSPACE via a two-sided, paired Wilcoxon test. In **i** and **k**, concordance was assessed by Pearson correlation and linear regression, with 95% confidence intervals indicated in **i**. A two-sided *t*-test was used to assess whether each correlation result was significantly non-zero. Adjustments for multiple comparisons were made in **f** using the Benjamini–Hochberg method.

To evaluate performance on real ST datasets, we next examined primary tumor specimens from three types of solid malignancy: melanoma, breast cancer and colon cancer. In total, six scRNA-seq/ST combinations, encompassing six bulk ST samples (*n* = 4 Visium; *n* = 2 legacy ST), including one HER2^+^ formalin-fixed, paraffin embedded (FFPE) breast tumor specimen and three scRNA-seq datasets from matching tumor subtypes, were analyzed^34–37^ (Supplementary Tables 1 and 3). All cell types in each scRNA-seq dataset were aligned by CytoSPACE (Fig. 2a and Supplementary Fig. 5) and compared to Tangram and CellTrek (Supplementary Fig. 5). CytoSPACE was highly efficient, processing a Visium-scale dataset in approximately 5 minutes, on average, with a single CPU core (Supplementary Table 4). This was true regardless of whether we applied shortest augmenting path or integer programming approximation approaches, both of which achieved similar results (Supplementary Table 5). To quantitatively compare the recovery of cell states with respect to spatial localization patterns in the tumor microenvironment (TME), we dichotomized assigned cells into two groups within each cell type by their proximity to tumor cells. We then assessed whether gene sets marking TME cell states with known localization were skewed in the expected orientation (Fig. 2b and Methods).

We started by considering T cell exhaustion, a canonical state of dysfunction arising from prolonged antigen exposure in tumor-infiltrating T cells^38^. Consistent with expectation, CytoSPACE recovered spatial enrichment of T cell exhaustion genes^39^ in CD4 and CD8 T cells mapped closest to cancer cells in all six scRNA-seq and ST dataset combinations (Fig. 2c,d, Supplementary Fig. 6a and Supplementary Tables 6 and 7). In contrast, Tangram and CellTrek produced single-cell mappings with substantially lower enrichment of T cell exhaustion genes in the expected orientation, with 25% to 33% of cases showing enrichment in the opposite direction, away from the tumor core (Fig. 2d, Supplementary Fig. 6a and Supplementary Tables 6 and 7).

**Extended Data Fig. 7:**
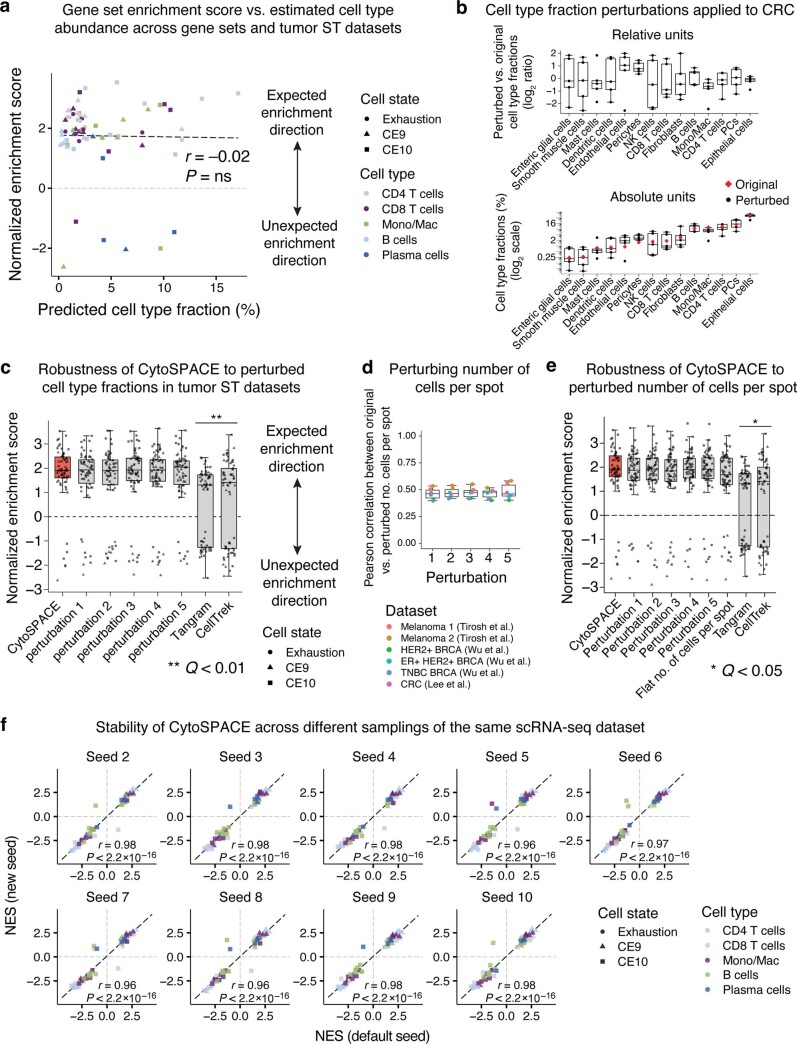
Robustness of CytoSPACE applied to tumor ST datasets. **a**, Same as Extended Data Fig. 5a but analyzing inferred cell type abundances vs. mean CytoSPACE performance across six tumor ST datasets (related to Fig. 2d and f), where performance is defined as cell state enrichments measured by normalized enrichment score (NES). Of note, to unify the expected enrichment directions, NES values for CE10 were multiplied by –1. **b**, Same as Extended Data Fig. 6a but showing cell type fraction perturbations for a representative CRC ST dataset (same as in Fig. 2a). **c**, Same as Fig. 2d and f but showing the impact of perturbing cell type fractions on CytoSPACE performance. **d**, Box plots showing Pearson correlations between perturbed and original estimates of the number of cells per spot for all six tumor ST datasets across five trials. **e**, CytoSPACE performance on all six tumor scRNA-seq/ST dataset pairs before and after perturbing estimates of the number of cells per spot across five trials (related to d) along with ‘flattening’ the number of cells per spot, in which spots were assigned the same number of cells. Group comparisons in panels c and e were performed using a two-sided paired Wilcoxon test for each CytoSPACE result versus each method in Fig. 2f, with Tangram and CellTrek shown as representative examples. P-values were corrected using the Benjamini-Hochberg method and expressed as q-values. *Q < 0.05; **Q < 0.01. Of note, the q-value in panels c and e is inclusive of all comparisons between CytoSPACE results and comparator methods in Fig. 2f. The box center lines, box bounds, and whiskers in b – e indicate the medians, first and third quartiles and minimum and maximum values within 1.5× the interquartile range of the box limits, respectively. **f**, Same as Fig. 2d and f but comparing NES values for cell state enrichment between the default seed and 9 additional random samplings of the scRNA-seq query dataset.

**Extended Data Fig. 8:**
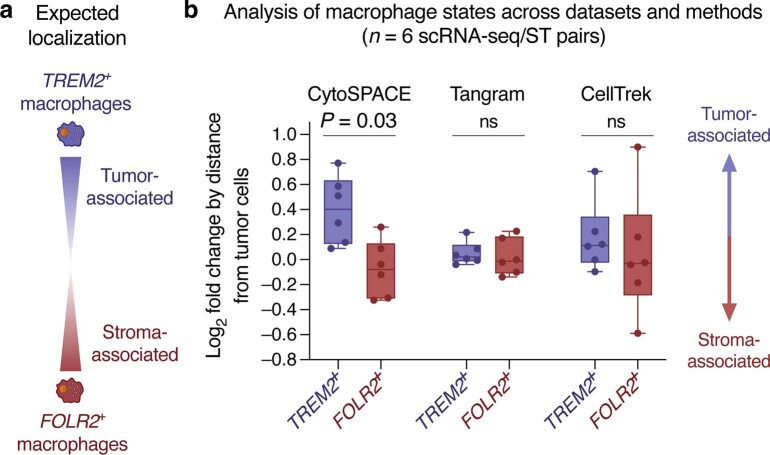
Single-cell spatial analysis of *TREM2*^+^ and *FOLR2*^+^ macrophage states across datasets and methods. **a**, Expected spatial localization of *TREM2*^+^ and *FOLR2*^+^ macrophages in human tumors (Nalio Ramos et al.). **b**, Box plots comparing the log_2_ fold change of *TREM2* and *FOLR2* expression in single macrophage/monocyte transcriptomes grouped into ‘near’ (Euclidean distance to tumor = 0) and ‘far’ (Euclidean distance to tumor > 0) categories, as described in Methods. Each point represents an scRNA-seq/ST pair analyzed in Fig. 2e. Single-cell mappings for each of the three methods are identical to Fig. 2. The box center lines, box bounds, and whiskers denote the medians, first and third quartiles and minimum and maximum values, respectively. Two-group comparisons were performed using a two-sided paired Wilcoxon test (indicated by the horizontal line above each pair of *TREM2*^+^ and *FOLR2*^+^ boxes). ns, not significant.

To demonstrate applicability to other spatially biased cell states, we next extended our analysis to diverse TME lineages, identifying cell-type-specific genes that vary in expression as a function of distance from tumor cells. To validate our results, we considered two recently defined cellular ecosystem subtypes in human carcinoma, CE9 and CE10 (ref. ^4^). These ‘ecotypes’, which were also observed in melanoma, each encompass B cells, plasma cells, CD8 T cells, CD4 T cells and monocytes/macrophages with stereotypical spatial localization. CE9 cell states are preferentially localized to the tumor core, whereas CE10 states are preferentially localized to the tumor periphery^4^. Using marker genes specific to each state^4^ (Supplementary Table 6), we asked whether single cells mapped by each method were consistent with CE9-specific and CE10-specific patterns of spatial localization. Indeed, as observed for T cell exhaustion factors, CytoSPACE successfully recovered expected spatial biases in CE9 and CE10 cell states across lymphoid and myeloid lineages (Fig. 2e), outperforming 12 previous methods in both the magnitude and orientation of marker gene enrichments (Fig. 2f, Supplementary Fig. 6 and Supplementary Table 7). Furthermore, consistent with simulation experiments, CytoSPACE results remained robust to perturbations of its input parameters (Extended Data Fig. 7). As further validation, we analyzed predicted spatial localization patterns of *TREM2*^+^ and *FOLR2*^+^ macrophages, which were recently shown to localize to the tumor stroma and to the tumor mass, respectively, across diverse cancer types^6^ (Extended Data Fig. 8a). Compared to Tangram and CellTrek, only CytoSPACE recapitulated these prior findings with statistical significance (Extended Data Fig. 8b). Moreover, when inferred spatial locations (close to tumor versus far from tumor) were projected onto uniform manifold approximation and projection (UMAP) embeddings of scRNA-seq data, single cells generally failed to cluster on the basis of their distance from tumor cells (Supplementary Fig. 7). These data underscore the ability of CytoSPACE to accurately identify spatially resolved cell states, including those not discernible from scRNA-seq or ST data alone.

**Extended Data Fig. 9:**
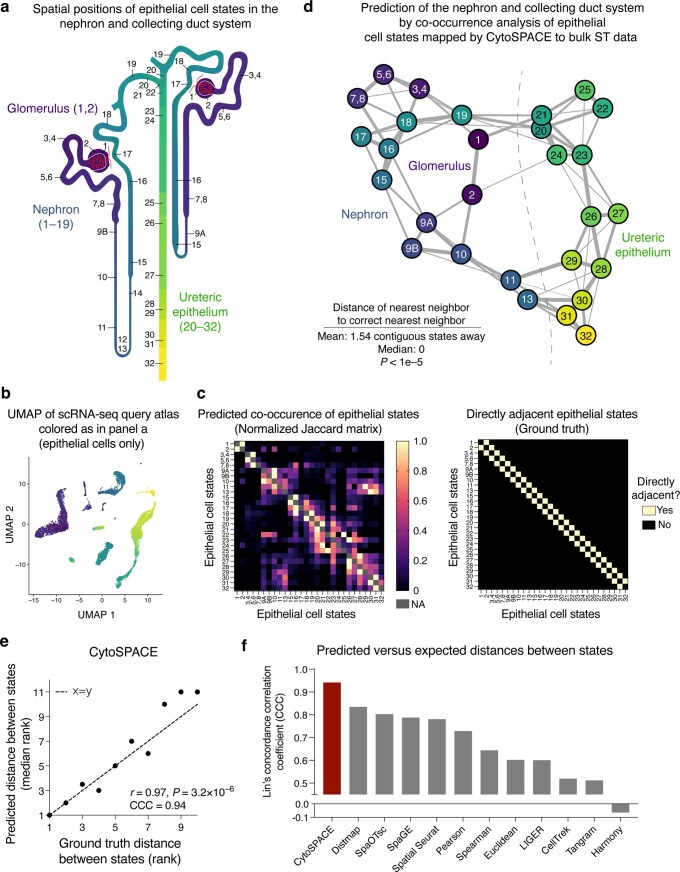
CytoSPACE-guided reconstruction of the nephron and collecting duct system. **a**, Similar to Fig. 2g but showing epithelial cell states colored by physically adjacent phenotypes. The corresponding cell state ontology is provided in Supplementary Table 8. **b**, UMAP embedding of a normal mouse kidney scRNA-seq atlas (mapped by CytoSPACE) and colored as in panel a (Methods). **c**, *Left:* Heat map showing the pairwise spatial overlap between all kidney epithelial cell states mapped by CytoSPACE to a 10x Visium sample of normal mouse kidney (related to Fig. 2h bottom). Overlap was determined by the Jaccard index and normalized to the maximum value per row (Methods). Of note, states 12 and 14 were imputed with zero abundance and not mapped by CytoSPACE. Self-comparisons are denoted by NA. *Right:* Heat map showing known adjacent states (as in panel a). **d**, Spring layout of the data in panel c, where each cell state is plotted along with its closest 4 neighbors (in rank space) inferred by CytoSPACE. Selected kidney structures are indicated. Edge thickness is proportional to the degree of overlap in rank space. Statistical significance was calculated by a one-sided permutation test, as described in Methods. **e**, Scatter plot comparing (i) the distance between each state *i* and the *nth* nearest neighbor (state *j*) predicted by CytoSPACE (median rank across all evaluable states, y-axis) with (ii) the distance between state *i* and its ground truth *nth* nearest neighbor (x-axis). Distances between states were calculated as the number of known consecutive states between *i* and *j*. Nearest neighbors from 1 to 10 were evaluated. Agreement was assessed by Pearson correlation and Lin’s concordance correlation coefficient (CCC). A two-sided t-test was applied to determine if the correlation coefficient was significantly non-zero. **f**, Same analysis as in panel e but for all evaluated methods, comparing performance using CCC. DEEPsc assigned all cells to the same spot and was omitted (Methods).

To further demonstrate how CytoSPACE can illuminate spatial biology, we explored two additional scenarios. First, we asked whether CytoSPACE can uncover densely packed cellular substructures in bulk ST data. For this purpose, we selected normal mouse kidney, which has highly granular spatial architecture. After mapping a well-annotated scRNA-seq atlas with more than 30 spatially resolved subtypes of kidney epithelium^40^ to a 10x Visium profile of normal mouse kidney^41^ (55-µm diameter per spot) (Fig. 2g and Supplementary Table 8), we assessed whether CytoSPACE recapitulates known patterns of spatial organization. Indeed, CytoSPACE (1) reconstructed known zonal regions (Fig. 2h, top, and Supplementary Fig. 8a); (2) identified cell types that preferentially co-localize to the glomerulus (~70-µm diameter^42^; Supplementary Fig. 8b); and (3) arranged nearly 30 epithelial states in spots consistent with their known locations in the nephron epithelium and collecting duct system^40^, outperforming previous methods (Fig. 2h, bottom, Fig. 2i and Extended Data Fig. 9).

**Extended Data Fig. 10:**
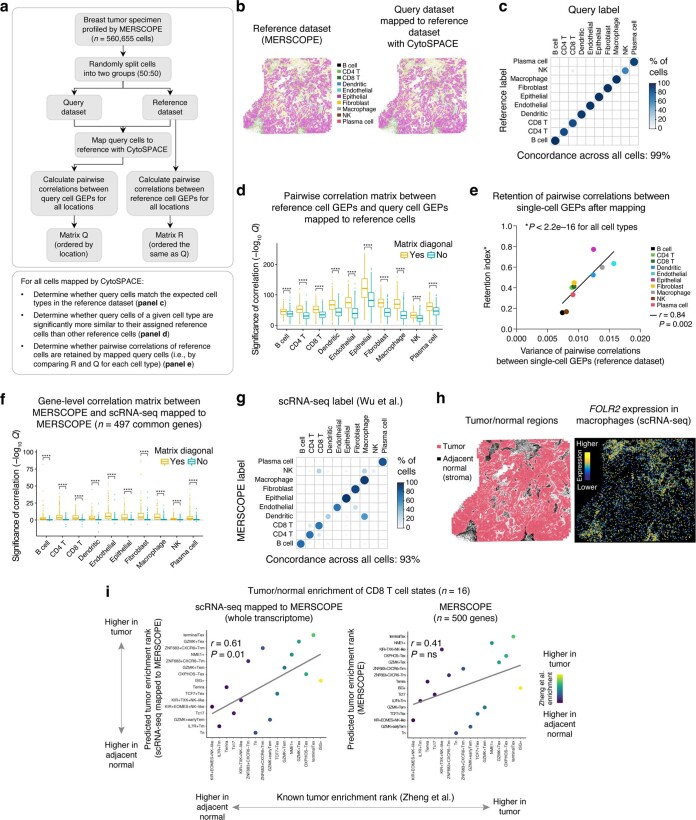
Technical assessment of CytoSPACE applied to single-cell ST data. **a**, Workflow for analyses in panels b–e. **b**, *Left*: MERSCOPE reference profile of a breast cancer specimen, with major cell types distinguished by color. *Right:* MERSCOPE query dataset mapped to the reference profile by CytoSPACE, with query cell types distinguished by color. **c**, Concordance of phenotypes between reference and query cells following alignment. **d**, Analysis of mapping accuracy, showing the significance of the Pearson correlation between the log_2_ GEPs of (i) the reference cells and (ii) query cells mapped to the reference cells, stratified by cell type. The matrix diagonal captures comparisons between query cell GEPs and their corresponding reference cell assignments. Non-matching pairwise combinations (off-diagonal entries) represent cell-type-specific controls. **e**, Analysis of the retention of pairwise distances between cells after mapping with CytoSPACE. For each cell type, the scatter plot shows a Retention index, defined as the Pearson correlation between matrices *Q* and *R*, versus the variance in matrix *R* (panel a). The significance of the linear regression line was assessed by a two-sided t-test. **f–i**, Extended analysis related to Fig. 2j, k. **f**, Analysis of gene-level concordance, showing the significance of the Pearson correlation between the log_2_ expression levels of (i) the scRNA-seq data (Wu et al.) mapped to MERSCOPE and (ii) the original MERSCOPE data, analyzed separately for each gene (*n* = 497 in common) and cell type. As a control, non-matching pairwise combinations of the same 497 genes were also assessed (off-diagonal entries in the correlation matrix). **g**, Concordance of cell type labels between MERSCOPE and scRNA-seq following alignment. **h**, *Left:* Tumor and adjacent normal regions determined as described in Methods. *Right*: *FOLR2* expression in single-cell transcriptomes (Wu et al.) annotated as ‘Macrophages/Monocytes’ and mapped by CytoSPACE, showing elevated levels in adjacent normal regions, consistent with expectation. **i**, Same as Fig. 2k but for CD8 T cells. In d and f, a maximum of 1,000 cells and 1,000 off-diagonal correlations per cell type were randomly sampled for analysis. For each cell type, p-values were Benjamini-Hochberg adjusted and expressed as –log_10_ q-values, which were multiplied by –1 for negative correlations. Group comparisons in d and f were evaluated using a one-sided Wilcoxon test relative to the matrix diagonal and p-values were Benjamini-Hochberg adjusted. ****Q < 0.0001. In d and f, the center lines, bounds, and whiskers indicate the medians, first and third quartiles and minimum and maximum values within 1.5× the interquartile range of the box limits, respectively. GEP, gene expression profile.

Finally, we asked whether CytoSPACE can enhance single-cell ST datasets with low gene throughput. To do so, we analyzed a breast cancer specimen with more than 550,000 annotatable cells and 500 pre-selected genes profiled by MERSCOPE (Vizgen) (Methods). First, we confirmed that CytoSPACE could accurately map single cells profiled by MERSCOPE and recapitulate their spatial dependencies (Extended Data Fig. 10a–e). Next, we mapped an scRNA-seq breast cancer atlas^37^ to the same MERSCOPE dataset. In addition to observing strong inter-platform agreement for most annotated cell types (Fig. 2j and Extended Data Fig. 10f,g), we confirmed striking biases in cancer-associated T cell signatures enriched in tumor or adjacent normal tissue^43^ (Fig. 2k, Extended Data Fig. 10h,i and Supplementary Table 9). Such enrichments were markedly more correlated with expected enrichments^43^ than those calculated from MERSCOPE data alone (Fig. 2k, Extended Data Fig. 10i and Supplementary Table 9). Collectively, these data emphasize the versatility of CytoSPACE for complex tissue reconstruction at the single-cell level.

In summary, CytoSPACE is a tool for aligning single-cell and spatial transcriptomes via global optimization. Unlike related methods, CytoSPACE ensures a globally optimal single-cell/spot alignment conditioned on a correlation-based cost function and the number of cells per spot. Moreover, it can be readily extended to accommodate additional constraints, such as the fractional composition of each cell type per spot (as inferred by RCTD^18^ or cell2location^27^, for example). In contrast, CellTrek is dependent on the co-embedding learned by Spatial Seurat, which can erase subtle yet important biological signals (for example, cell state differences), as was recently shown^44^. Although Tangram is robust in idealized settings, it cannot guarantee a globally optimal solution. Although CytoSPACE requires two input parameters, both parameters can be reasonably well estimated using standard approaches, suggesting that they are unlikely to pose a major barrier in practice. Furthermore, on both simulated and real datasets, CytoSPACE was substantially more accurate than related methods. As such, we anticipate that CytoSPACE will prove useful for deciphering single-cell spatial variation and community structure in diverse physiological and pathological settings.

## Methods

### CytoSPACE analytical framework

CytoSPACE leverages linear optimization to efficiently reconstruct ST data using single-cell transcriptomes from a reference scRNA-seq atlas. To formulate the assignment problem mapping individual cells in scRNA-seq data to spatial coordinates in ST data, let an *N* × *C* matrix *A* denote single-cell gene expression profiles with *N* genes and *C* cells; let an *M* × *S* matrix *B* denote gene expression profiles (GEPs) of ST data with *M* genes and *S* spots; and let *G* be the vector of length *g* that contains the subset of desired genes shared by both datasets. For both GEP matrices, values are first normalized to counts per million (CPM) (or transcripts per million for platforms covering the full gene body) and then transferred into log_2_ space. Thus, in its default implementation, CytoSPACE uses all genes as input and does not involve a dimension reduction step. Next, we estimate (by default) the number *n*_*s*_, *s* = 1,⋯,*S*, of cells contributing RNA content in the *s*th spot of ST data (see ‘Estimating the number of cells per spot’). We assume that the *s*th spot contains *n*_*s*_ sub-spots that can each be assigned to a single cell and build an *M* × *L* matrix $\overline B$ by replicating the *s*th column of *B*, *n*_*s*_ times, where $L = \mathop {\sum}\nolimits_{s = 1}^S {n_s}$ denotes the total number of estimated sub-spots in the ST data. As described in the following sections, we then sample the scRNA-seq matrix *A* such that the total number of cells, with cell types represented according to their inferred fractional abundances, matches the total number of columns in $\overline B$, yielding an *N* × *K* matrix $\overline A$, where *K = L*. Next, define an assignment *x* := [*x*_*kl*_], 0 ≤ *x*_*kl*_ ≤ 1, *k* = 1,⋯,*K* and *l* = 1,⋯,*L*, where *x*_*kl*_ denotes the assignment of the *k*th cell in the scRNA-seq data to the *l*th sub-spot in the ST data. Of note, although *x*_*kl*_ is only explicitly constrained to real values within this range, a globally optimal solution will naturally satisfy $x_{kl} \in \left\{ {0,1} \right\}$. We find the optimal cell/sub-spot assignment x* that minimizes the following linear cost function:

$$x^ \ast = \arg \min Cost\left( x \right) = \arg \min \mathop {\sum}\limits_{k = 1}^K {\mathop {\sum}\limits_{l = 1}^L {d_{kl}x_{kl}} } ,$$

subject to:

$$\mathop {\sum}\limits_{l = 1}^L {x_{kl} = 1,k = 1, \cdots ,K} ,\mathop {\sum}\limits_{k = 1}^K {x_{kl} = 1,l = 1, \cdots ,L,}$$

where *d*_*kl*_ denotes the distance between the GEPs of the *k*th cell and the *l*th sub-spot. The above constraints guarantee that each cell is assigned only to one sub-spot, and each sub-spot receives only one cell. In general, *d*_*kl*_ can be obtained using any metric that quantifies the similarity between the GEPs of the reference and target datasets. We examined different similarity metrics for simulated data and selected Pearson correlation as below due to its computational efficiency:

$$d_{kl} = - corr\left( {\bar A_k^G,\bar B_l^G} \right),$$

where $\overline A _k^G$ and $\overline B _l^G$ denote the *k*th and *l*th columns of expression matrices $\overline A$ and $\overline B$, respectively, for the shared genes in *G*.

We provide two possible solvers for CytoSPACE, both of which will return the globally optimal solution of the above problem as formulated. The first of these implements the shortest augmenting paths-based Jonker–Volgenant algorithm, in which we solve the dual problem of the above formulation defined as:

$${{{\mathrm{max}}}}\left( {\mathop {\sum}\limits_{k = 1}^K {u_k} + \mathop {\sum}\limits_{l = 1}^L {v_l} } \right),$$

subject to:

$$r_{kl}: = d_{kl} - \left( {u_k + v_l} \right) \ge 0,l = 1, \cdots ,L,k = 1, \cdots ,K,$$

where for the dual variables *u*_*k*_ and *v*_*l*_, the reduced cost *r*_*kl*_ is defined as *d*_*kl*_ − (*u*_*k*_ + *v*_*l*_). The dual problem reformulates our optimization task to find an alternative reduction of the cost function with maximum sum and non-negative reduced costs. In summary, this algorithm constructs the auxiliary network (or, equivalently, a bipartite graph) and determines from an unassigned row *k* to an unassigned column *j* an alternative path of minimal total reduced cost and uses it to augment the solution^26^. In practice, despite time complexity *O*(*L*^3^), the Jonker–Volgenant algorithm is substantially faster than most available algorithms for solving the assignment problem. By default, CytoSPACE calls the lapjv solver from the lapjv software package (version 1.3.14) in Python 3, which makes use of AVX2 intrinsics for speed (https://github.com/src-d/lapjv)^26^. With this solver, CytoSPACE runs in approximately 5 minutes, on average, using a single core on a 2.4-GHz Intel Core i9 chip for a standard 10x Visium sample with an estimated average of five cells per spot.

We provide an alternate solver based on the cost scaling push–relabel method^31^ using the Google OR-Tools software package in Python 3. This solver is an integer programming approximation method in which exact costs are converted to integers with some loss of numerical precision and which runs with time complexity *O*(*L*^2^ log (*LC*)), where *C* denotes the largest magnitude of an edge cost. In practice, this solver is approximately as fast as the Jonker–Volgenant-based solver detailed above. However, for very large numbers of cells to be mapped, it can offer faster runtimes. Furthermore, it is supported more broadly across operating systems, so we recommend this solver for users working on systems that do not support AVX2 intrinsics as required by the lapjv solver. For users who want to obtain the exact results of lapjv on operating systems that do not support the lapjv package, an equivalent but considerably slower solver implementing the Jonker–Volgenant algorithm is provided via the ‘lap’ package (version 0.4.0), which has broad compatibility.

### Estimating cell type fractions

To overcome variability in cell type fractional abundance between a given ST sample and a reference scRNA-seq dataset, the first step of CytoSPACE requires estimating cell type fractions in the ST sample (Fig. 1a). Of note, only global estimates for the entire ST array are required, and these may be obtained by combining spot-level fractions by cell type. Although an intriguing future extension of CytoSPACE would be to estimate cell type fractions as part of the optimization routine, many deconvolution methods have been proposed to determine cell type composition from ST spots^14,20,27,28^, and any such method can be deployed for this purpose. In this study, we used Spatial Seurat^14^ from Seurat version 3.2.3 for our primary analyses, and we show that correlations between estimated and true fractions of distinct cell types are high in simulated data (Extended Data Fig. 3a). After loading raw count matrices, we performed SCTransform() and RunPCA() with default parameters followed by FindTransferAnchors() in which the pre-processed scRNA-seq and ST data served as the reference and query, respectively. We then obtained spot-level predictions by TransferData() and obtained global predictions by summing prediction scores per cell type across all spots and scaling the sum of cell type scores to 1.

In addition to Spatial Seurat, we tested the performance of RCTD^18^ for estimating global cell type fractions as input to CytoSPACE (Supplementary Fig. 3). RCTD version 2.0.0 (package spacexr in R) was employed with doublet_mode = ‘full’ and otherwise default parameters to obtain cell type fraction estimates per spot, followed by summing spot-normalized result weights per cell type across all spots and scaling the sum to 1.

### Estimating the number of cells per spot

The number of detectably expressed genes per cell (‘gene counts’) tightly corresponds to total captured mRNA content, as measured by the sum of unique molecular identifiers (UMIs) per cell^45^. As gene counts are routinely used as a proxy for doublets or multiplets in scRNA-seq experiments, we hypothesized that the sum of UMIs per ST spot may reasonably approximate the number of cells per spot, as required for the second step of CytoSPACE (Fig. 1a). To test this hypothesis while blunting the effect of outliers, technical variation and the impact of cell volume^46^, we first normalized UMIs to CPM per spot and then performed log_2_ adjustment. We then estimated the number of cells per ST spot by fitting a linear function through two points. For the first point, we assumed that the minimum number of cells per spot is 1 and that this minimum in cell number corresponds to the minimum sum of UMIs in log_2_ space. For the second point, we assumed that the mean number of cells per spot corresponds to the mean sum of UMIs in log_2_ space and set this value according to user input. For 10x Visium samples in which spots generally contain 1–10+ cells per spot, we employed a mean of five cells per spot throughout this work. For legacy ST samples with larger spot dimensions, we selected a mean of 20 cells per spot. The number of cells for every spot was calculated from this fitted function. In support of our hypothesis, for simulated ST datasets, we found that the Pearson correlation between the estimated and real number of cells ranged between 0.80 and 0.93, depending on the dataset and spot resolution evaluated, with log_2_ adjustment outperforming the sum of UMIs in the original linear scale (that is, without CPM) (Extended Data Fig. 3b–d). The same was true when comparing against the number of cells per spot analyzed by cell segmentation (VistoSeg^29^) applied to previously analyzed imaging data from a mouse brain Visium sample (Extended Data Fig. 3e), further validating our approach. Although this estimation component is provided by default, users may also provide their own estimates for this step, including those generated by cell segmentation methods (for example, VistoSeg^29^ and Cellpose^30^).

### Harmonizing the number of cells per cell type

The third step of CytoSPACE equalizes the number of cells per cell type between the query scRNA-seq dataset and the target ST dataset (Fig. 1a). This is accomplished by sampling the former to match the predicted quantities in the latter using one of the following methods:

#### Duplication

Let *num*_*sc,k*_ and *num*_*ST,k*_ denote the real and estimated number of cells per cell type *k* in scRNA-seq and ST data, respectively. For cell type *k*, if *num*_*sc,k*_ < *num*_*ST,k*_, CytoSPACE retains all available cells in the scRNA-seq data and also randomly samples *num*_*ST,k*_ − *num*_*sc,k*_ cells from the same *num*_*sc,k*_ cells. Otherwise, it randomly samples *num*_*ST,k*_ from the *num*_*sc,k*_ available cells with cell type label *k* in the scRNA-seq data. By default, CytoSPACE applies this method for real data to ensure that all cells assigned are biologically appropriate.

#### Generation

Here, when *num*_*sc,k*_ < *num*_*ST,k*_, instead of duplicating cells, new cells of a specific type are generated with independent random gene expression levels by sampling each gene from the gene expression distribution of cells of the same type uniformly at random. We used this method for benchmarking simulations to avoid bias in measuring precision owing to the presence of duplicated cells (Fig. 1b–d, Extended Data Figs. 4–6 and Supplementary Figs. 2–4).

### Simulation framework

To evaluate the accuracy and robustness of CytoSPACE (Fig. 1b), we simulated ST datasets with known single-cell composition using previously annotated Slide-seq datasets of mouse cerebellum and hippocampus sections^32^. Let *Sl* be an *M* × *B* gene expression matrix of a Slide-seq puck with *M* genes and *B* beads. To create a higher gene coverage version of *Sl*, denoted *Sc*, we used previously annotated scRNA-seq datasets of the same brain regions^33^ to replace *Sl* beads with single-cell transcriptomes. After quality control, in which outlier cells with more than 1,500 genes were removed, we matched each bead in the Slide-seq datasets with the nearest cell of the same cell type in the scRNA-seq dataset by Pearson correlation. We did this separately for each mouse brain region. As single cells may be matched with more than one bead, to obtain unique single-cell transcriptomes we permuted genes between cells of the same cell type. For each cell, we replaced 20% of its transcriptome, with genes randomly selected per cell, with that of another randomly selected cell of the same cell type such that the latter is not a duplicate of the former. For simplicity, we matched the number of beads present in the two tissues by randomly sampling beads from the hippocampus data down to the number present in the cerebellum data.

Having created an *Sc* matrix for each brain region, we next sought to generate ST datasets with defined spot resolution. For this purpose, we imposed an *m* × *n* spatial grid over the entire puck. In each grid spot *x*_*ij*_, i = 1,…,*n*, j = 1,…,m, we calculated the sum of raw counts $\overline {Sc} _{ij}$ of the cells located within the grid spot *x*_*ij*_. Because the spatial resolution of ST data varies depending on the technology used, we simulated ST datasets with an average of 5, 15 and 30 cells per spot.

Finally, to (1) leverage the scRNA-seq data underlying each *Sc* matrix as a query dataset and (2) emulate technical variation between platforms, we added noise to the scRNA-seq data in defined amounts. To this end, we selected a percentage of genes *p* to perturb and then randomly selected a corresponding subset of genes from each cell to which noise was added from the exponentiated Gaussian distribution 2^*N*(0,1)^. We considered noise perturbations for the following values of *p*: 5%, 10% and 25%. Despite the addition of noise, UMAP plots of perturbed transcriptomes remained similar to the original data, implying maintenance of biologically realistic data structure (Extended Data Fig. 2b–e).

### Quality control considerations for cell-to-spot alignment

There are two key scenarios in which mismatch between scRNA-seq and ST data can occur. In the first scenario, cell types are detectable in the scRNA-seq dataset but not in the spatial dataset. CytoSPACE addresses this issue by requiring cell type abundance estimates as input (for example, using Seurat^14^, RCTD^18^ or cell2location^27^). In doing so, cell types missing from the ST dataset will generally be omitted from the spatial mapping (if imputed with zero fractional abundance) or inferred with low fractional abundance, minimizing their impact on performance.

In the second scenario, cell types are detectable in the spatial dataset but not in the scRNA-seq dataset, leading to incorrect mapping. Except for cell types that are either rare or prone to dissociation-induced losses, this scenario is uncommon, as droplet sequencing can readily canvas all major cell types in a given tissue sample. Other methods for spatial spot decomposition, including Seurat^14^, RCTD^18^ and cell2location^27^, have the same limitation, which is usually negligible in practice.

Although the Jonker–Volgenant algorithm is guaranteed to optimally solve the assignment problem given its cost function, there is no underlying probabilistic framework for estimating mapping uncertainty. An alternative is to determine whether a given cell type belongs to a given spatial spot *after* mapping—that is, whether a spot contains at least one cell of the same cell type. Notably, this definition is considerably less demanding than the metric described in the ‘Performance assessment’ subsection below. Nevertheless, to explore this possibility, we implemented the following procedure. First, to identify the top marker genes for each cell type mapped by CytoSPACE, we sequentially applied NormalizeData(), ScaleData() and FindAllMarkers() from Seurat version 4.0.1 to the scRNA-seq query dataset using default parameters. We then normalized and scaled the ST dataset using the same workflow. For each cell type *i* with at least five, and up to 50, marker genes (denoted by *m*) identified by –log_10_-adjusted *P* value with log_2_ fold change >0, we randomly selected 50 spatial spots for which CytoSPACE assigned at least one cell of cell type *i* and 50 spatial spots without at least one cell of cell type *i*. If fewer than 50 spots satisfied a given condition, we sampled 50 spots with replacement. Next, we used cell-to-spot assignments to reconstitute each selected spot as a pseudo-bulk transcriptome from the normalized and scaled scRNA-seq dataset by averaging over the assigned cells. We subsequently trained a support vector machine (e1071 version 1.7.8 in R) to distinguish the two groups of pseudo-bulks from the previous step using the top *m* marker genes of cell type *i*. With this model, we calculated the probability, termed a confidence score, that cell type *i* belongs to each spot in the normalized and scaled ST dataset. Finally, for each mapped cell of type *i*, we retrieved its spot-specific confidence score.

We evaluated this approach on simulated ST data where ground truth is known (Supplementary Fig. 1a). Although the fraction of incorrectly mapped cells (defined as above) was already low before applying this filter (<5%), it successfully distinguished correctly mapped cells from incorrectly mapped cells with high statistical significance, with nearly all areas under the curve (AUCs) exceeding 0.8 for classifying individual cell types (Supplementary Fig. 1b,c). Moreover, at a confidence threshold above 10%, virtually every correctly mapped cell was retained, whereas more than 75% of incorrectly mapped cells were removed (Supplementary Fig. 1d,e). Thus, this procedure, which is available via the CytoSPACE GitHub repository, may be used as an optional post-processing step for exploring alignment quality.

### Benchmarking analysis with simulated datasets

To fully evaluate the performance of CytoSPACE, we performed an extended benchmarking analysis including Tangram, CellTrek and ten additional methods that may be adapted for our use case (Extended Data Fig. 4). In considering which methods to include, we required methods that (1) are applicable to a single-cell query dataset and spatial reference dataset, including bulk ST data; (2) produce an output, or involve an intermediate step, in which the two datasets are aligned, allowing imputation of single-cell spatial coordinates in the query dataset (for example, scRNA-seq integration techniques, some gene imputation methods and naive distance metrics); and (3) are peer reviewed with a publicly available software implementation.

Many previous methods for ST analysis fail to satisfy these requirements, including methods designed for spot-level decomposition (for example, cell2location^27^ and RCTD^18^; Extended Data Fig. 1), spatial clustering (for example, BayesSpace^47^) and spatial coordinate prediction without a spatial reference (for example, novoSpaRc^13^). Accordingly, our benchmarking analysis consists of three dedicated cell-to-spot mapping methods (CytoSPACE, Tangram and CellTrek); three single-cell integration methods (Harmony^48^, LIGER^49^ and Seurat version 3 (ref. ^14^)); four methods from which cell-to-spot assignments can be extracted (DistMap^50^, SpaGE^51^, DEEPsc^52^ and SpaOTsc^53^); and three naive methods (Pearson correlation, Spearman correlation and Euclidean distance). Below we describe the application of each approach.

#### CytoSPACE

For each ST resolution and scRNA-seq noise level, we estimated the fractional abundance of known cell types in the ST sample via Spatial Seurat, as described in the ‘Estimating cell type fractions’ subsection. We then ran CytoSPACE with the ‘generated cells’ option and with the lapjv solver implemented in Python (package lapjv, version 1.3.14).

#### Tangram

Like CytoSPACE and in contrast to the other methods considered here, Tangram seeks to arrange input cells across spots optimally, and cell-to-spot mappings for each input cell are strongly inseparable from the cell-to-spot mappings of other cells. Thus, to ensure a fair comparison with CytoSPACE, we ran Tangram (version 1.0.2) with the same input cells mapped by CytoSPACE, including cells newly generated after resampling to match predicted cell type numbers. We also provided a normalized vector of CytoSPACE’s cell number per spot estimate as the density prior (density_prior argument). We trained Tangram on CPM-normalized scRNA-seq data in two ways: (1) using all available genes per cell and (2) using the top marker genes stratified by cell type. To identify marker genes using Seurat (version 4.1.0), we applied NormalizeData() with default parameters and FindAllMarkers() with only.pos = TRUE, min.pct = 0.1 and logfc.threshold = 0.25. The top 100 genes by average log_2_ fold change were then selected for each cell type.

#### CellTrek

Given that CellTrek heavily duplicates input cells (by default) and also filters input cells based on whether mutual nearest neighbors are identified between cells and spots^25^, we provided CellTrek (version 0.0.0.9000) with all cells present in each simulated ST dataset (without the newly generated cells mapped by CytoSPACE and Tangram). After single cells were assigned to spatial coordinates, we selected the closest ST spot for each cell via Euclidean distance. As the CellTrek wrapper does not handle ST input without associated h5 and image files, we modified the code to accommodate ST datasets from other sources. CellTrek was run with default parameters, with the exception of (1) limiting the repel functionality (repel_r = 0.0001), as this parameter forces imputed spatial coordinates to arbitrarily deviate from their original predictions, and (2) setting spot_n to twice the mean number of cells per spot for each spatial resolution tested.

#### DistMap

DistMap seeks to computationally reconstruct ST data at single-cell resolution from paired scRNA-seq. It uses marker genes and a binarization approach calculating Matthews correlation coefficients to obtain distributed positional assignments for each cell^50^.

For our benchmarking, we provided DistMap (version 0.1.1) with all input cells and spots, restricting genes to marker genes (selected as described for benchmarking Tangram with top genes) expressed in at least five cells and five spots. Count matrices were CPM normalized and log_2_ adjusted. After creation of a DistMap object with the normalized ST data provided for the insitu argument, we binarized the scRNA-seq data via binarizeSingleCellData(dm, seq(0.15, 0.5, 0.01)) per author recommendations. We prepared a binarized version of the ST data matrix by setting all non-zero counts to 1 and then replaced the insitu.matrix member variable of the DistMap object with this binarized version. We performed the cell-to-spot mapping with mapCells() and assigned each cell to the spot with the highest score as returned in the mcc.scores member variable.

#### SpaOTsc

SpaOTsc is a method for inferring spatial properties of scRNA-seq data, designed primarily for the investigation of spatial cell–cell communications^53^. As the first step in this process, SpaOTsc computes a map between single cells and a spatial dataset using an optimal transport approach on marker genes.

For our benchmarking, we provided SpaOTsc (version 0.2) with all input cells and spots, restricting genes to marker genes (selected as described for benchmarking Tangram with top genes) expressed in at least five cells and five spots. Following tutorial instructions, we implemented SpaOTsc as follows. We first normalized counts to sum to 10,000 per cell or spot, respectively, and then log_2_ transformed the resulting scRNA-seq (df_sc) and ST (df_is) matrices. From the normalized scRNA-seq data, we performed principal component analysis (PCA) with prcomp in R and then computed the Pearson correlation coefficient matrix (sc_pcc) between single cells from the top 40 principal components. To obtain a Matthews correlation coefficient matrix (mcc) between cells and spots, we binarized each normalized data matrix (resulting in df_sc_bin and df_is_bin for scRNA-seq and ST matrices, respectively) with a quantile threshold of 0.7 and then computed the Pearson correlation coefficient over all cell–spot pairs. We then ran SpaOTsc with the following set of commands: C = np.exp(1-mcc), issc = SpaOTsc.spatial_sc(sc_data = df_sc, sc_data_bin = df_sc_bin, is_data = df_is, is_data_bin = df_is_bin, sc_dmat = np.exp(1-sc_pcc), is_dmat = is_dmat), out = issc.transport_plan(C**2, alpha = 0.1, rho = 100.0, epsilon = 1.0, cor_matrix = mcc, scaling = False). Each cell was then assigned to the spot with the highest score as returned in the output of issc.transport_plan().

#### DEEPsc

DEEPsc is a deep-learning-based method for imputing spatial information onto scRNA-seq data given a spatial reference atlas^52^. DEEPsc first transfers the spatial reference atlas data to a space of reduced dimensionality via PCA and then performs network training over it. The scRNA-seq data are projected into the same PCA space and fed into the DEEPsc network, which outputs a matrix of likelihoods that each cell originated from each spot in the ST tissue.

For our benchmarking, we provided DEEPsc (version number not available; last GitHub commit when cloned: 5 June 2022) with all input cells and spots, with each input matrix CPM normalized and then log transformed via log1p and with genes restricted to those present in both matrices. DEEPsc was run with 50,000 iterations in parallel mode for training as previously described^52^ and with otherwise default parameters.

#### SpaGE

SpaGE, or Spatial Gene Enhancement using scRNA-seq, is a method for increasing gene coverage in ST measurements by integrating spatial data with higher-coverage scRNA-seq datasets^51^. SpaGE uses the domain adaptation algorithm PRECISE to project datasets into a shared space, in which gene expression predictions are then computed through a k-nearest neighbors approach. Although SpaGE was designed for gene expression prediction rather than mapping cells to spots, as it includes an integration step, it is possible to use this integration space for cell-to-spot mapping.

To do so while making full use of the SpaGE framework (version number not available; last GitHub commit when cloned: 20 July 2021), we added to the source code a command to return the single nearest spot neighbor for each cell in the SpaGE integrated space. We then provided the modified SpaGE code with all input cells and spots. Following the tutorial recommendations, we excluded genes not expressed in at least ten cells and then CPM normalized and log_2_ transformed the scRNA-seq matrix while normalizing the ST matrix to median counts per spot, followed by log_2_ transformation. SpaGE was run with n_pv = 30, again per tutorial recommendations, and otherwise default parameters.

#### Spatial Seurat

Seurat, a well-known method for integrating single-cell expression datasets that works by identifying ‘anchors’ between datasets, can be used with spatial data as well^14^. We tested Spatial Seurat integration for assigning cells to spots using Seurat version 3. After loading scRNA-seq and ST count matrices into Seurat objects, we pre-processed both with SCTranform() and then used the standard integration protocol of FindTransferAnchors(normalization.method = ‘SCT’), followed by TransferData(). Cell-to-spot assignments were determined by the predicted.id returned from the resulting predictions assay.

#### Harmony

Harmony is a method for integrating multiple scRNA-seq datasets into a joint embedding space, employing clustering methods over principal component representations of the data to obtain linear correction factors for integration^48^. As a dataset integration method, Harmony does not provide direct cell-to-spot mapping results. Thus, for our benchmarking, we used the method to first integrate the full single-cell and corresponding spatial datasets and then assigned each cell to its nearest spot within the integration space by selecting the spot with minimum Euclidean distance to the cell.

To obtain the integration space representations, we followed the standard Harmony protocol. We first merged Seurat objects created from the scRNA-seq and ST count matrices and then applied the standard Seurat processing pipeline of NormalizeData(), FindVariableFeatures(), ScaleData() and RunPCA(), all with default parameters. With the resulting Seurat object, we ran Harmony version 0.1 with group.by.vars = ‘orig.ident’ and otherwise default parameters.

#### LIGER

Like Harmony, LIGER is another method designed for single-cell expression dataset integration^49^, although LIGER relies instead on an integrative non-negative matrix factorization approach to embed features in a low-dimensional space, incorporating both dataset-specific and shared factors. As described above for Harmony, we used LIGER to obtain a shared embedding space between the scRNA-seq and ST datasets and then assigned cells to spots according to minimum Euclidean distance.

To run LIGER (version 1.0.0), we created a LIGER object and then processed it with package functions normalize(), selectGenes(var.thresh = 0.2) and scaleNotCenter(), for normalization, gene selection and scaling, respectively, and then applied online_iNMF() and quantile_norm() to align the datasets following the tutorial^49^. All parameters not specified here were set to defaults. Embeddings were extracted from the LIGER object member variable H.norm.

In addition to the above methods, we tested Euclidean distance (calculated with the spatial.distance.cdist function of scipy version 1.8.0), Pearson correlation and Spearman correlation. Here, each cell was assigned to the spot that either minimized distance (Euclidean distance) or maximized correlation (Pearson and Spearman correlations). All ground truth cells were evaluated without resampling, and input datasets were CPM normalized and log_2_ adjusted before analysis.

#### Performance assessment

To determine the accuracy of single-cell mapping (Fig. 1d, Extended Data Figs. 4–6 and Supplementary Figs. 2–4), we classified assigned locations that exactly matched ground truth spots as correct. Letting $TP_{sc}$ denote the number of correct assignments, we defined single-cell precision (*Pr*_*sc*_) as

$$Pr_{sc} = \frac{{TP_{sc}}}{{No.\,unique\,mapped\,cells\,with\,ground\,truth\,locations}}$$

Of note, because generated cells (see the ‘Harmonizing the number of cells per cell type’ subsection) did not have a corresponding ground truth location, they were excluded from this calculation. Separately, although CellTrek can assign the same cell ID *i* to multiple spots, any cell of ID *i* mapped to the correct spot at least once was considered correct. This was done without inflating the denominator or penalizing incorrect mappings for other cells with ID *i*.

### Measuring robustness of CytoSPACE in simulation

To be broadly useful, a computational method such as CytoSPACE must exhibit robustness to reasonable variation or error in inputs. With this in mind, we tested CytoSPACE’s consistency and robustness to variation across input parameters.

#### Robustness to cell fraction estimation error

To mimic realistic technical error in estimating cell type fractions, in which proportionally larger error can be expected for rarer cell types, we introduced multiplicative noise within a four-fold range, with noise inversely dependent upon the original fraction estimate. First, for each cell type *i* in a sample, we randomly sampled *y*_*i*_ from a Gaussian distribution with mean zero and standard deviation inversely dependent on the original fraction estimate *x*_*i*_ for cell type:

$$\sigma = \frac{1}{{2x_i^{1/3}}},y_i \sim N\left( {0,\sigma ^2} \right)$$

Here, the cubic root smooths the distribution toward the four-fold perturbation range desired. To restrict the range strictly to within a four-fold perturbation, we imposed a maximum absolute value of 2 on the resulting value:

$$z_i = {{{\mathrm{max}}}}\left( { - 2,\min \left( {2,y_i} \right)} \right)$$

The perturbation of each original estimate was then computed as

$$\overline {x_i} = x_i \cdot 2^{z_i}$$

with the resulting values then renormalized to unit sum.

We tested CytoSPACE with this noise model in simulation with five replicates for each simulated test case (see the ‘Simulation framework’ subsection), evaluating results via single-cell assignment precision as described in the ‘Performance assessment’ subsection (Extended Data Fig. 6a,b).

#### Robustness to cell number per spot estimation error

We introduced noise to estimates of number of cells per spot with a similar protocol to that described above for perturbing cell type fraction estimates. First, for each spot in a sample, we randomly sampled *y*_*i*_ from a Gaussian distribution with mean zero and standard deviation inversely dependent on the original estimate *n*_*i*_ for cell type *i*:

$$\sigma = \frac{p}{{n_i^{1/3}}},y_i \sim N\left( {0,\sigma ^2} \right)$$

In the above distribution, *p* denotes a tuning parameter that we set by spatial resolution in such a way as to produce similar Pearson correlations between the original and perturbed estimate as we observed between the CytoSPACE estimate, based on RNA content, and the VistoSeg estimate, based on image segmentation (within the range of 0.50–0.55; Extended Data Fig. 3e). To achieve this, we set *p* to 1.4 (simulated data with estimated mean of five cells per spot), 1.7 (simulated mouse cerebellum data with estimated mean of 15 cells per spot), 2.2 (simulated mouse cerebellum data with estimated mean of 30 cells per spot), 2.6 (simulated mouse hippocampus data with estimated mean of 15 cells per spot) and 3.7 (simulated mouse hippocampus data with estimated mean of 30 cells per spot).

To restrict the range of values to a feasible region, we imposed a minimum number of cells per spot of 1 and a maximum number of cells per spot of 110% of the original maximum *M*. The perturbed values $\overline {n_i}$ were, thus, computed as

$$\overline {n_i} = \max \left( {1,\min \left( {n_i \cdot {{{\mathrm{Round}}}}\left( {2^{y_i}} \right),1.1M} \right)} \right)$$

We tested CytoSPACE with this noise model in simulation with five replicates for each simulated test case (see ‘Simulation framework’ subsection), evaluating results via single-cell assignment precision as described in the ‘Performance assessment’ subsection (Extended Data Fig. 6c–e).

#### Robustness to sampling variation

Although most steps of the algorithm are deterministic, CytoSPACE requires that the input scRNA-seq dataset be resampled to create a pool of cells matching those expected in the ST dataset; this sampling is done at random. To test consistency of results across different samples, we ran CytoSPACE ten times with different seeds for each simulation case described in the ‘Simulation framework’ subsection. Single-cell precision of the assignment was calculated as described above (‘Performance assessment’ subsection). Results for this analysis are shown in Supplementary Fig. 4a.

#### Robustness to distance metric

In addition to Pearson correlation, the default distance metric that we implement for CytoSPACE, we tested CytoSPACE performance with alternative distance metrics Spearman correlation and Euclidean distance as shown in Supplementary Fig. 4b. For each ST resolution and scRNA-seq noise level in simulated data (as described in the ‘Simulation framework’ subsection), we ran CytoSPACE with Spearman correlation and Euclidean distance substituted for the distance metric.

### ST datasets for TME community analysis

Melanoma ST data generated by Thrane et al.^35^ were downloaded from https://www.spatialresearch.org/resources-published-datasets/doi-10-1158-0008-5472-can-18-0747/. Pre-processed ST datasets of breast cancer (Visium fresh-frozen and FFPE) and colorectal cancer (CRC) (fresh-frozen) specimens were downloaded from 10x Genomics (https://www.10xgenomics.com/spatial-transcriptomics/). Annotations of regions containing tumor cells were downloaded from 10x Genomics for the Visium FFPE breast cancer sample and shared by 10x Genomics upon request for the Visium fresh-frozen breast cancer sample analyzed in this work. A pre-processed Visium array of a fresh-frozen triple-negative breast cancer (TNBC) specimen (1160920F) was obtained from Wu et al.^37^ along with tumor boundaries. Additional details are available in Supplementary Table 1.

### scRNA-seq tumor atlases

All analyzed tumor scRNA-seq data, which were downloaded as pre-processed count (UMI-based) or transcript (non-UMI-based) matrices (Supplementary Table 1), were selected and curated to clinically match the ST specimens analyzed in this work (see the ‘Molecular classification of breast cancer specimens’ subsection). Additionally, author-supplied annotations were used for all scRNA-seq reference datasets analyzed in Fig. 2 (detailed in Supplementary Table 1), with the following modifications. For the melanoma dataset generated by Tirosh et al.^36^, we excluded normal melanocytes and divided T cells into CD4 and CD8 subsets by the expression of *CD8A/CD8B* and *CD4/IL7R*, respectively, as previously described^4^. For the breast cancer dataset from Wu et al.^37^ and for the CRC dataset from Lee et al.^34^, the authors’ annotations were mapped to cell types according to the scheme in Supplementary Table 3. Of note, we excluded T cells that could not be confidently classified as CD8 or CD4 T cells and myeloid cells that could not be confidently classified as monocytes/macrophages or dendritic cells.

### Molecular classification of breast cancer specimens

When available, author annotations were used to determine estrogen receptor (ER) and human epidermal growth factor receptor 2 (HER2) enrichment status for each scRNA-seq and ST tissue breast cancer sample. For the FFPE breast cancer specimen from 10x Genomics without receptor status annotation, we examined the expression of *ESR1* (ER) and *ERBB2* (HER2) genes. We reclassified the FFPE breast cancer ST specimen as HER2^+^/ER^−^ based on high expression of *ERBB2* without appreciable *ESR1* expression.

### Mapping of single-cell transcriptomes onto tumor ST samples

For the analyses in Fig. 2a–f, Extended Data Figs. 7 and 8 and Supplementary Figs. 5 and 6, CytoSPACE and the other benchmarking methods described in the ‘Benchmarking analysis with simulated datasets’ subsection were applied as follows:

#### CytoSPACE

Cell type fractions were computed using Spatial Seurat (see the ‘Estimating cell type fractions’ subsection), and CytoSPACE was run with the ‘duplicated cells’ option and the lapjv solver as implemented in the lapjv Python package on a single CPU core. For all Visium samples, we set the mean number of cells per spot to 5, whereas, for legacy ST samples (melanoma ST data), we set this parameter to 20.

#### Tangram

As input, we analyzed the same single-cell transcriptomes mapped by CytoSPACE, including duplicates, along with a density prior (density_prior argument) determined by the number of cells per spot estimated by CytoSPACE. Because Tangram performed best with all genes when used for simulated ST datasets (Fig. 1d, Extended Data Fig. 4 and Supplementary Figs. 2 and 3), we ran Tangram (version 1.0.2) on CPM-normalized scRNA-seq data with 24 CPU cores on all available genes. Other parameters were set to default.

#### CellTrek

Given CellTrek’s internal filtering mechanism (see the ‘Benchmarking analysis with simulated datasets’ subsection), we provided all cells in the corresponding scRNA-seq atlases as input (without duplication or downsampling). For Visium samples, we ran CellTrek (version 0.0.0.9000) with default parameters with 24 CPU cores (reduction = ‘pca’, intp = T, intp_pnt = 10,000, intp_lin = F, nPCs = 30, ntree = 1,000, dist_thresh = 0.4, top_spot = 10, spot_n = 10, repel_r = 5, repel_iter = 10, keep_model = T) and then assigned cells from raw output coordinates to their nearest spot by Euclidean distance. For the legacy ST samples (melanoma), we modified the code to handle inputs without h5 and image files, as detailed above. To fit the larger spot resolution in the legacy ST datasets, we fixed spot_n to 40. Other parameters were the same as above.

#### Other methods

The other benchmarking methods (DistMap, SpaOTsc, DEEPsc, SpaGE, Spatial Seurat, Harmony, LIGER, Euclidean distance, Pearson correlation and Spearman correlation) were implemented according to the details described in their corresponding sections in ‘Benchmarking analysis with simulated datasets’, with the following exception: for computational feasibility over especially large scRNA-seq datasets, we ran SpaOTsc for two scRNA-seq/ST pairs (CRC and TNBC) with the protocol described above for ‘Tangram’, providing the cells mapped by CytoSPACE rather than the entire scRNA-seq dataset.

### Running time analysis

To evaluate the efficiency of CytoSPACE in practice and benchmark against recent dedicated cell-to-spot mapping methods, we recorded running times for CytoSPACE, Tangram and CellTrek across all scRNA-seq tumor atlas/ST pairs tested (*n* = 4 pairs with Visium ST data, *n* = 2 pairs with lower-resolution legacy ST data) (Supplementary Table 4) with parameter details as described above. For CytoSPACE, we report running times for both exact (shortest augmenting path via the lapjv solver) and integer approximation solvers and both with and without a Spatial Seurat pre-processing step for obtaining input cell type fractional abundances. Data loading and file writing steps were excluded from running times for all methods. Methods were tested on similar, although not identical, systems, with CytoSPACE, Spatial Seurat pre-processing steps and Tangram tested on a computing cluster providing Intel E5-2640v4 (2.4-GHz base and 3.4-GHz max frequencies, with an associated 128 GB RAM), Intel 5118 (2.3 GHz base and 3.2 GHz max frequencies, with an associated 191 GB of RAM) and AMD 7502 (2.5-GHz base and 3.35-GHz max frequencies, with an associated 256 GB of RAM) processors and with CellTrek tested on a server with an Intel E5-2680v3 processor and an associated 230 GB of RAM. With the exception of CytoSPACE, in which the core mapping function uses only a single core, all methods were provided with 24 cores.

### Validation of alternative solver

To verify that the integer approximation solver we provide as a fast alternative to the recommended exact solver (lapjv) yields similar results, we measured the proportion of single cells mapped to the same location across the two solver methods. For each scRNA-seq tumor atlas/ST pair tested, we mapped the same single cells after pre-processing for duplication and downsampling to match the estimated cell type fractions in tissue via CytoSPACE with exact and integer approximation solvers, and we report the percentage of cells mapped to the same spot in each method (Supplementary Table 5). For duplicated cells, no distinction was made between the copies.

### Spatial enrichment analysis

To determine whether single cells mapped to ST spots showed enrichment of known spatially resolved gene expression programs, cells were first partitioned into two groups (‘close’ and ‘far’) based on their distance from cancer cells. For breast cancer ST samples, all of which were profiled by 10x Visium, we used tumor boundary annotations determined by a pathologist to group cells. For melanoma and CRC datasets, the mean Euclidean distance of each TME cell to the nearest five tumor cells (mapped by the respective alignment method) was determined. For the melanoma dataset, melanoma cells were considered as tumor cells, whereas, in the CRC dataset, tumor epithelial cells were considered for the purpose of identifying tumor locations in tissue. For each TME cell type, the resulting distances were median stratified into ‘close’ and ‘far’ groups. This was done for two main reasons. First, the CRC sample lacked tumor boundary annotations. Second, although melanoma datasets included such annotations, the low spatial resolution of the legacy ST platform prevented precise co-registration with spatial spots at the tumor–stroma interface.

To quantify spatial enrichment, we used pre-ranked gene set enrichment analysis (GSEA) implemented in fgsea (version 1.14.0) with nperm = 10,000. As input, all spatially mapped single-cell transcriptomes were loaded by cell type into Seurat version 4.1.0 (min.cells = 5) and normalized with NormalizeData(). For each method and cell type, we then generated a gene list ranked by log_2_ fold change for the identity classes ‘near’ and ‘far’ using FoldChange(). If fewer than ten cells of a cell type were assigned to spots within one partition by at least one method, we excluded that cell type from the enrichment analysis. Of note, several methods (SpaOTsc, DEEPsc, Seurat, Hamony and Euclidean distance) failed to map all evaluated cell types to regions both closer to and farther from tumor cells, precluding the use of GSEA (as described below in the ‘Spatial enrichment analysis’ subsection) on the affected cell types. In such cases, statistical comparisons to CytoSPACE were performed excluding the affected cell types. As CytoSPACE and Tangram were each run with the same scRNA-seq input, before running Seurat and fgsea we performed random sampling of cells mapped by all other methods to match the number of cells per cell type mapped by CytoSPACE and Tangram and to ensure a fair comparison among methods. This was done as described in ‘Harmonizing the number of cells per cell type—Duplication’. Gene sets for T cell exhaustion and CE9/CE10-associated cell states were derived by Zheng et al.^39^ and Luca et al.^4^, respectively. All evaluated gene sets and underlying GSEA results are provided in the supplement (Supplementary Tables 6 and 7, respectively).

### Measuring robustness of CytoSPACE on real data

We repeated the robustness testing described previously in ‘Measuring robustness of CytoSPACE on simulated data’ with real data, applying CytoSPACE under various perturbations to the task of spatial enrichment analysis in TME samples and quantifying performance according to the recovery of expected spatial enrichments of gene sets in the TME as described in ‘Spatial enrichment analysis’ (Extended Data Fig. 7). The perturbation analyses were conducted in the same manner as with simulated data, except for the robustness to cell number per spot estimation error analysis, for which the tuning parameter *p* was set for scRNA-seq/ST dataset pairs as follows: 1.4 (Visium data), 1.9 (legacy ST data, melanoma slide 2) and 2.3 (legacy ST data, melanoma slide 1).

### Spatially resolved macrophage states

To evaluate the spatial localization of *TREM2*^+^ and *FOLR2*^+^ macrophages^6^ (Extended Data Fig. 8), single-cell transcriptomes annotated as ‘macrophages/monocytes’ were mapped to ST spots as described above (‘Mapping of single-cell transcriptomes onto tumor ST samples’; Supplementary Table 1) and ordered based on their spatial distance (Euclidean) from tumor cells. All cells were processed with Seurat as described in ‘Spatial enrichment analysis’. To calculate distance, we used the same metric described for melanoma and CRC datasets (‘Spatial enrichment analysis’). For cells mapped within tumor boundaries annotated by a pathologist (breast cancer datasets), distances were set to zero. We then divided cells into ‘near’ (distance = 0) and ‘far’ (distance > 0) groups and calculated the log_2_ fold change of each gene using FoldChange() in Seurat (Extended Data Fig. 8b).

### Integrative single-cell spatial analysis of healthy mouse kidney

For the analyses presented in Fig. 2g–i, Extended Data Fig. 9 and Supplementary Fig. 8, we downloaded (1) a well-annotated scRNA-seq atlas encompassing immune cells, stromal elements and more than 30 spatially resolved subtypes of kidney epithelium^40^ and (2) a 10x Visium sample of normal mouse kidney^41^ (Supplementary Table 1). Kidney epithelial cell states lacking a numeric identifier (as in Fig. 2g) were omitted, and states corresponding to the same phenotype were merged (3 and 4, 5 and 6, 7 and 8; Fig. 2g). The datasets were subsequently aligned with CytoSPACE as described in ‘Mapping of single-cell transcriptomes onto tumor ST samples’ but with the mean number of cells per spot set to 10. Using epithelial cells, which have ground truth locations in the scRNA-seq atlas, we analyzed the following zonal regions: cortex (outermost region), outer medulla (central region) and inner medulla (innermost region), with the outer medulla further subdivided into the outer stripe (proximal to the cortex) and inner stripe (proximal to the inner medulla) (Fig. 2h, top, and Supplementary Fig. 8a).

We established a ground truth rank for each epithelial cell state, reflecting its relative distance to epithelial state 32 (‘deep medullary epithelium of pelvis’), which corresponds to the base of the ureteric epithelium (UE) in the inner medulla as previously reported^40^ (Fig. 2g and Supplementary Table 8). Then, using single-cell spatial coordinates determined by CytoSPACE, we calculated the mean Euclidean distance of each epithelial cell state to the centroid of epithelial cells mapped to epithelial state 32. Regardless of whether we examined nephron or UE, correlations between predicted and ground truth distances were high, demonstrating CytoSPACE’s potential for granular mapping (Fig. 2i).

For the analysis in Extended Data Fig. 9, we tested whether CytoSPACE can resolve the known structure of the nephron and UE collecting system (Extended Data Fig. 9a), which is not discernible from the scRNA-seq atlas (Extended Data Fig. 9b) or ST dataset^41^ alone. For this purpose, we scored spatial spots as 1 if at least one cell of a given cell type was mapped by CytoSPACE and 0 otherwise. We then converted the resulting binary square matrix, with cell types as rows and cell types as columns, into a Jaccard similarity matrix J that quantifies spatial overlap among epithelial states (Extended Data Fig. 9c, left). After filtering all but the four nearest neighbors of each epithelial state in J, we converted each row to rank space and created an undirected graph from the data using igraph version 1.2.6 in R. We then visualized the graph using layout_with_fr(), the Fruchterman and Reingold force-directed layout algorithm implemented in igraph (Extended Data Fig. 9d). To determine statistical significance (Extended Data Fig. 9d), we devised a permutation approach in which we first determined the nearest neighbor *N*_*i*_ of each epithelial state *i* in J. We then calculated the minimum number of physically adjacent epithelial states (denoted by *x*_*i*_) between *N*_*i*_ and the ground truth nearest neighbor(s) of *i* (Extended Data Fig. 9c, right). After calculating *x*_*i*_ for all evaluable epithelial states, the results were averaged, denoted $\overline x$. After this, we randomly permuted each row of J and recalculated the mean distance $\overline x ^\prime$. We repeated this for a total of 100,000 iterations to calculate the empirical *P* value of $\overline x$. To create the UMAP plot in Extended Data Fig. 9b, we sequentially applied the following Seurat version 4.0.1 commands to the log-normalized scRNA-seq data of epithelial cell states from Ransick et al.^40^: FindVariableFeatures() with selection.method = ‘vst’ and nfeatures = 2,000, ScaleData(), RunPCA(), FindNeighbors() with dims = 1:10 and RunUMAP() with dims = 1:30.

### Application to single-cell ST data

Although a major goal of CytoSPACE is reconstruction of bulk ST data at the single-cell level, it is also directly applicable to single-cell ST data. To do this efficiently for extremely large single-cell ST datasets, we implemented a sampling routine to uniformly partition single-cell ST datasets without replacement into bins of up to 10,000 cells each (by default), which balances considerations of cellular diversity and mapping efficiency. Specifically, the single-cell ST dataset is first randomly partitioned without replacement into *n* bins of 10,000 ST cells each. Next, for each bin (1,…,*n*), 10,000 single-cell transcriptomes are sampled from the scRNA-seq query dataset (by default) according to the procedure described in ‘Harmonizing the number of cells per cell type—Duplication’ above. Although the entire procedure is reproducible and anchored to a specific seed at initialization, the scRNA-seq dataset is newly resampled for each bin 1,…,*n* to promote robustness. Finally, CytoSPACE is run on each bin, and the results are combined to produce a single unified output.

For the analyses in Fig. 2j,k and Extended Data Fig. 10, a pre-processed MERSCOPE profile of an FFPE human breast cancer sample (HumanBreastCancerPatient1; Vizgen MERFISH FFPE Human Immuno-oncology Data Set, May 2022) was downloaded from Vizgen (https://vizgen.com/data-release-program/) (Supplementary Table 1). Cells with fewer than 100 transcripts and those with fewer than ten genes detected were excluded from the analysis, yielding 560,655 cells with 149 detected genes per cell, on average. The gene-by-cell count matrix was normalized by downsampling, which eliminated potential confounding factors such as cell volume, by normalizing the total transcripts per cell to be the same (300 transcripts per cell). Using Seurat version 4.1.1 to analyze the normalized data, we identified the top 100 variable genes using FindVariableFeatures() and clustered the cells with FindClusters() using resolution = 0.8. Leveraging canonical marker genes, clusters were annotated as fibroblasts (*COL1A1* or *COL5A1* high), endothelial cells (*PECAM1* or *VWF* high), macrophages (*FCGR3A* or *C1QC* high), dendritic cells (*CD1C* or *CD207* high), lymphocytes (*CD3E*, *TRAC*, *ZAP70*, *MS4A1*, *GNLY* or *MZB1* high) and epithelial (remaining). Lymphocytes were further clustered using the top 300 variable genes with resolution = 1.2 and annotated as CD4 T cells (*CD3E*, *TRAC*, *ZAP70* or *FOXP3* high and no *CD8A*), CD8 T cells (*CD3E*, *TRAC* or *ZAP70* high and *CD8A* high), natural killer (NK) cells (*GNLY* high and no *CD3E*), B cells (*MS4A1* high) and plasma cells (*MZB1* high); clusters that did not meet these conditions but showed strong expressions of non-lymphocyte markers were annotated accordingly using epithelial and stromal markers above.

To account for errors in transcript assignment arising from overlapping cells in the z-series, gene expression in the center z-plane (z = 3) was compared with expression in the peripheral z-plane (z = 0) for each segmented cell. Transcripts detected in either of the z-planes were first isolated as individual gene-by-cell count matrices. Then, all genes whose expression significantly differed between the two z-planes for one or more cell types were identified using a two-sided Wilcoxon test (nominal *P* < 0.05). For each of these genes, if expression was significantly higher in the center z-plane for one cell type but significantly higher in the z = 0 plane for another, the gene was considered a potential contaminant and set to 0 in all cells of the latter cell type.

For the analysis presented in Extended Data Fig. 10a–e, we began by randomly splitting the MERSCOPE dataset (50:50) into ‘scRNA-seq’ query and ST reference datasets (Extended Data Fig. 10a). We then mapped query cells to the reference as described above, running CytoSPACE with five CPU cores, the number of cells per spot set to 1 and the global fractional abundance of each cell type set to its proportion in the reference dataset (Extended Data Fig. 10b). We observed strong agreement for cell type labels (Extended Data Fig. 10c), and, for each cell type, the GEPs of mapped cells were more correlated with their assigned reference cells than with other reference cells of the same cell type (Extended Data Fig. 10d). We next asked whether pairwise transcriptomic distances between single cells were retained (Extended Data Fig. 10a). To do so for each evaluable cell type, we first calculated the pairwise correlation matrix *Q* of single-cell GEPs (in log_2_ space) in the scRNA-seq query dataset. This was done after assigning query cells to spatial locations in the reference. We then did the same for the reference dataset, yielding matrix *R*. Both matrices were ordered identically according to the same single-cell spatial coordinates, allowing us to determine whether the spatial correlation structure was recapitulated among mapped cells. Indeed, by calculating a retention index for each cell type, defined as the Pearson correlation between the two matrices, we observed highly significant retention of pairwise distances for each cell type (*P* < 2.2 × 10^−16^; Extended Data Fig. 10e). To ensure a fair assessment, before creating each matrix we sampled an equivalent number of cells per cell type (without replacement) based on the lowest common denominator in the reference dataset (*n* = 150 cells). We found that the degree of retention was proportional to the variance among GEPs in the reference dataset—that is, cell types with lower transcriptomic heterogeneity in the reference (that is, more uniform GEPs) had less spatial structure and lower retention of pairwise distances, consistent with expectation (Extended Data Fig. 10e).

As the MERSCOPE dataset lacked *ESR1* (estrogen receptor) and *PGR* (progesterone receptor) among the 500 target genes but showed elevated expression of *ERBB2* (encoding HER2), we selected HER2^+^ breast tumors profiled by scRNA-seq^37^ as the query dataset in Fig. 2j,k (Supplementary Tables 1 and 3). To ensure sufficient overlap in co-detected genes, we removed cells from the scRNA-seq dataset with fewer than 50 expressed genes (CPM > 0) overlapping the MERSCOPE panel. Next, we mapped the scRNA-seq atlas to the MERSCOPE sample, running CytoSPACE with five CPU cores, the number of cells per spot set to 1 and the global fractional abundance of each cell type set to its proportion as determined above.

To evaluate the spatial enrichment of cell states in Fig. 2j,k and Extended Data Fig. 10f–i, individual cells were first partitioned into two regions based on their Euclidean distance to epithelial cells. An epithelial cell was assigned to the tumor region if located within 100 µm of more than 50 epithelial cells. This threshold was selected based on a density-based analysis, where two major distributions of epithelial cell densities were observed, with ~50 epithelial cells per radius of 100 µm representing a local minimum between the two distributions. Then, of the remaining cells, a cell was assigned to the tumor region if located within 100 µm of a tumor epithelial cell; otherwise, it was assigned to the adjacent normal region (that is, stromal; Extended Data Fig. 10h). For the analyses presented in Fig. 2k and Extended Data Fig. 10i, the log_2_ fold change of each gene in tumor versus stromal regions was determined for CD4 and CD8 T cells with the raw MERSCOPE data (500 genes) or scRNA-seq data (whole transcriptome) mapped to MERSCOPE. Pre-ranked GSEA was applied as described in ‘Spatial enrichment analysis’ for the top 200 signature genes of each pan-cancer T cell state defined by Zheng et al.^43^ except for ‘CD4T_IL7R–Tn’, which lacked signature genes in the MERSCOPE dataset. For this analysis, fgsea package version 1.20.0 was used. Ground truth was determined as the rank of the log_2_ fold change between the tumor odds ratio and normal odds ratio of each evaluated T cell state, as reported in Supplementary Table 3 of Zheng et al.

### Statistics

All statistical tests were two-sided unless stated otherwise. The Wilcoxon test was used to assess statistical differences between two groups. Adjustment for multiple hypothesis testing was done via Benjamini–Hochberg where applicable. Linear concordance was determined by Pearson (*r*) correlation or Spearman (*ρ*) correlation, and a two-sided *t-*test was used to assess whether the result was significantly non-zero. All statistical analyses were performed using R versions 3.5.1 and 4.0.2+, Python 3.8, MATLAB_R2019a and Prism 9+ (GraphPad Software).

### Reporting summary

Further information on research design is available in the Nature Portfolio Reporting Summary linked to this article.

## Online content

Any methods, additional references, Nature Portfolio reporting summaries, source data, extended data, supplementary information, acknowledgements, peer review information; details of author contributions and competing interests; and statements of data and code availability are available at 10.1038/s41587-023-01697-9.

### Supplementary information

Supplementary Information Supplementary Figs. 1–8

Reporting Summary

Supplementary Table Supplementary Tables 1–9

## Data Availability

The publicly available expression datasets analyzed in this work (Supplementary Table 1) are available from the Gene Expression Omnibus with accession numbers GSE171406, GSE72056, GSE176078 and GSE132465; from Zenodo at https://zenodo.org/record/4739739#.YlL1A9NBzxg; from the Broad Institute Single Cell Portal at https://portals.broadinstitute.org/single_cell/study/slide-seq-study; from the Spatial Research Lab at https://www.spatialresearch.org/resources-published-datasets/doi-10-1158-0008-5472-can-18-0747/; from Vizgen at https://vizgen.com/data-release-program/; from 10x Genomics at https://support.10xgenomics.com/spatial-gene-expression/datasets/; and from GitHub at https://github.com/qinzhu/kidneycellexplorer/tree/master/data. Additional data supporting the findings in this work are available in the main text, figures, extended data and supplementary files.

## Code availability

CytoSPACE version 1.0 was coded in Python and used to generate the results in this work. It is available, along with documentation, vignettes and helper scripts for creating CytoSPACE inputs and for estimating cell type fractions, at https://github.com/digitalcytometry/cytospace. A user-friendly web portal for running CytoSPACE is available at https://cytospace.stanford.edu.
