# Supplementary material for: High-resolution alignment of single-cell and spatial transcriptomes with CytoSPACE
Source: Nat Biotechnol. 2023 Mar 6;41(11):1543–8. doi: 10.1038/s41587-023-01697-9 (PMC10635828; doi:10.1038/s41587-023-01697-9)
Supplement: Supplementary file 1 — Supplementary Figs. 1–8 [file 41587_2023_1697_MOESM1_ESM.pdf]

nature biotechnology

Brief Communication

https://doi.org/10.1038/s41587-023-01697-9

High-resolution alignment of single-cell and spatial transcriptomes with CytoSPACE

In the format provided by the authors and unedited

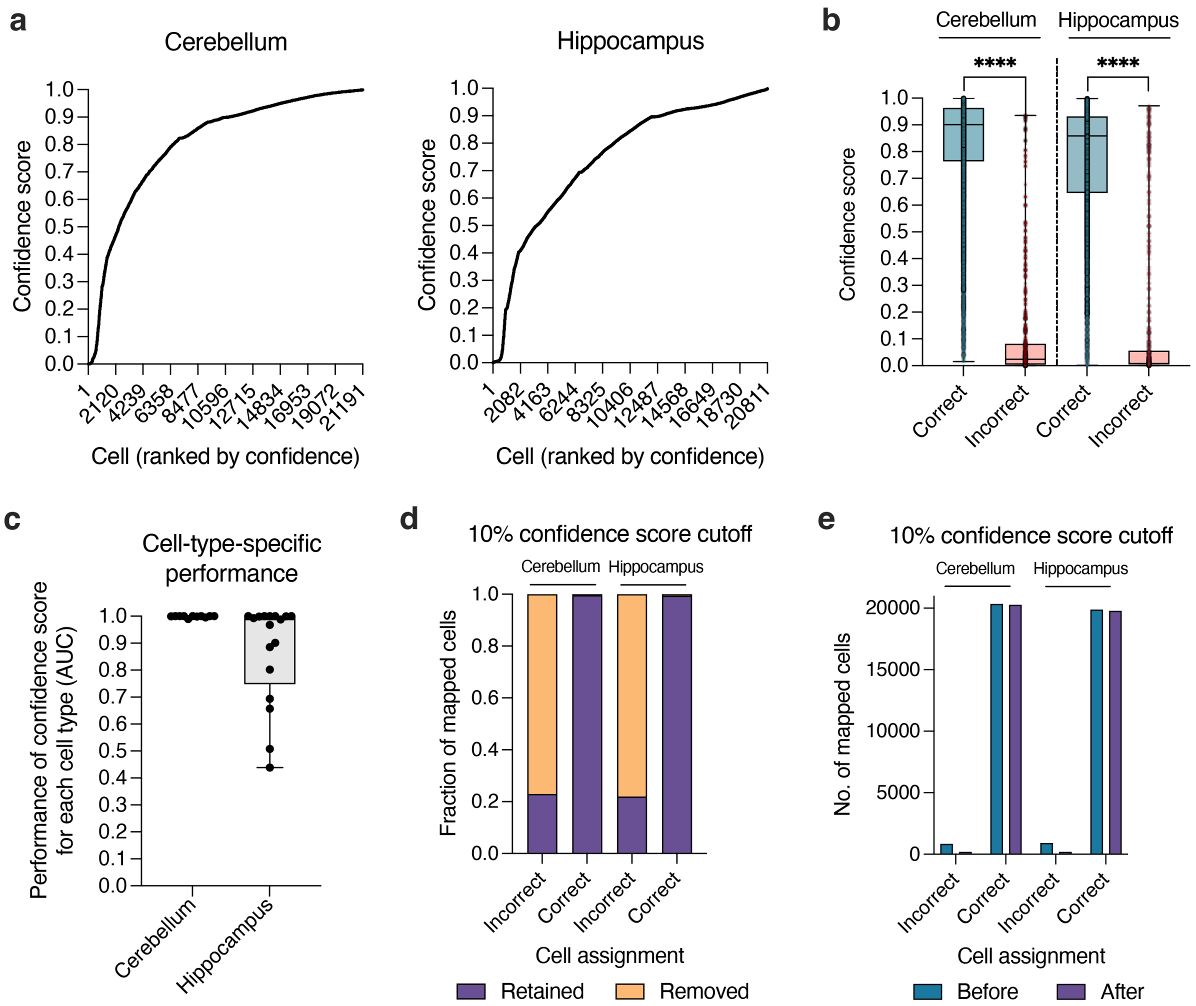

Supplementary Figure 1: Estimation of alignment uncertainty in simulated ST datasets. **a-e**, Application of support vector machine modeling to predict cell-to-spot mapping quality, expressed as "confidence scores," for scRNA-seq data mapped to simulated ST datasets of mouse cerebellum and hippocampus (mean of 5 cells per spot) using CytoSPACE. Single-cell RNA-seq query data were first perturbed with the addition of noise to 5% of each transcriptome (**Methods**). Details of the approach and datasets are supplied in **Methods**. **a**, Confidence scores for all mapped cells. **b**, Box plots showing confidence scores stratified by brain region and correct/incorrect assignments. For a given cell of type *i*, "correct" was defined as spots containing at least one cell of type *i*. Statistical significance was determined by a two-sided Wilcoxon test. ****$P < 2e\text{-}16$. **c**, Box plots showing the area under the curve (AUC) for distinguishing correct from incorrect spots by cell type ($n = 11$, cerebellum; $n = 17$, hippocampus). **d-e**, Impact of imposing a 10% confidence score threshold (>0.1) on the fraction (d) and absolute number (e) of retained cells. The box center lines, box bounds, and whiskers in panels b and c denote the medians, first and third quartiles and minimum and maximum values, respectively.

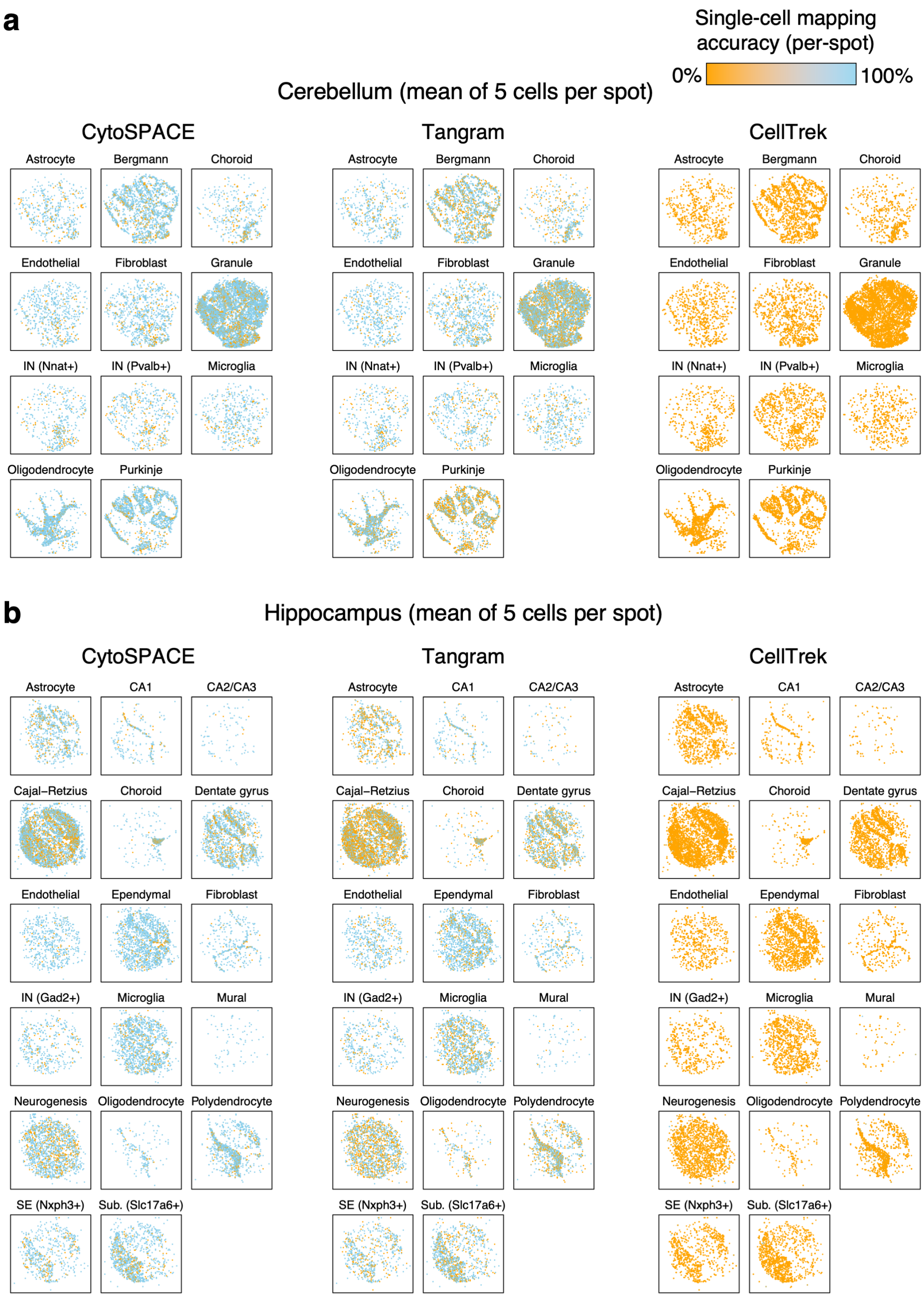

Supplementary Figure 2: CytoSPACE, Tangram, and CellTrek alignments for all cell types analyzed in simulated ST datasets (related to Fig. 1c). a-b, Heat maps depicting single-cell mapping accuracy, defined as the fraction of single cells correctly mapped to their ground truth spot (**Methods**), shown for three methods and all evaluated cell types mapped to mouse cerebellum (*n* = 11 cell types) (a) and hippocampus (*n* = 17 cell types) (b) ST datasets defined by simulation, with a mean of 5 cells per spot. CytoSPACE assignments, shown for single-cell transcriptomes with noise applied to 5% of the transcriptome (**Methods**), demonstrate strongest concordance with ground truth. IN, Interneuron; SE, Subiculum Entorhinal; Sub., Subiculum.

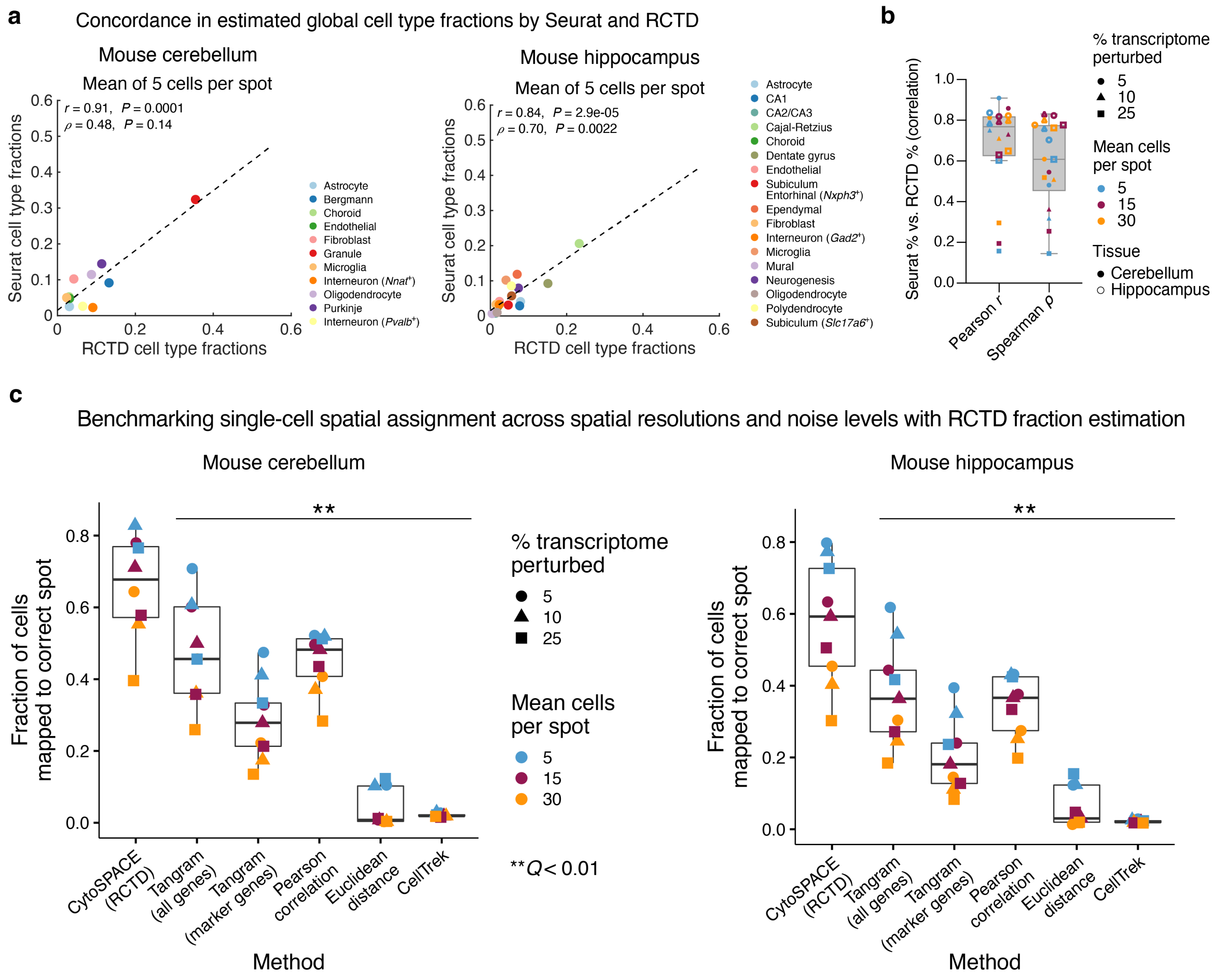

Supplementary Figure 3: Performance of CytoSPACE with RCTD. a-b, Comparison of cell type fractions estimated by Spatial Seurat and RCTD for (a) simulated datasets with a mean of 5 cells per spot and 5% noise added to scRNA-seq data and (b) simulated datasets across all analyzed spot resolutions and noise levels (**Methods**). Concordance was assessed by Pearson correlation (*r*), Spearman correlation (*ρ*), and linear regression (dashed lines). A two-sided t-test was used to assess whether each correlation result was significantly nonzero. **c,** Same as **Extended Data Figure 4** but showing the application of CytoSPACE with RCTD for cell type fraction estimation (rather than Spatial Seurat) against selected comparator methods (same as **Fig. 1d**). Raw data are provided in **Supplementary Table 2**. The box center lines, box bounds, and whiskers in b and c indicate the medians, first and third quartiles and minimum and maximum values within 1.5× the interquartile range of the box limits, respectively. Statistical significance was determined using a two-sided paired Wilcoxon test relative to CytoSPACE. P-values were corrected using the Benjamini-Hochberg method and are expressed as q-values (**$Q < 0.01$).

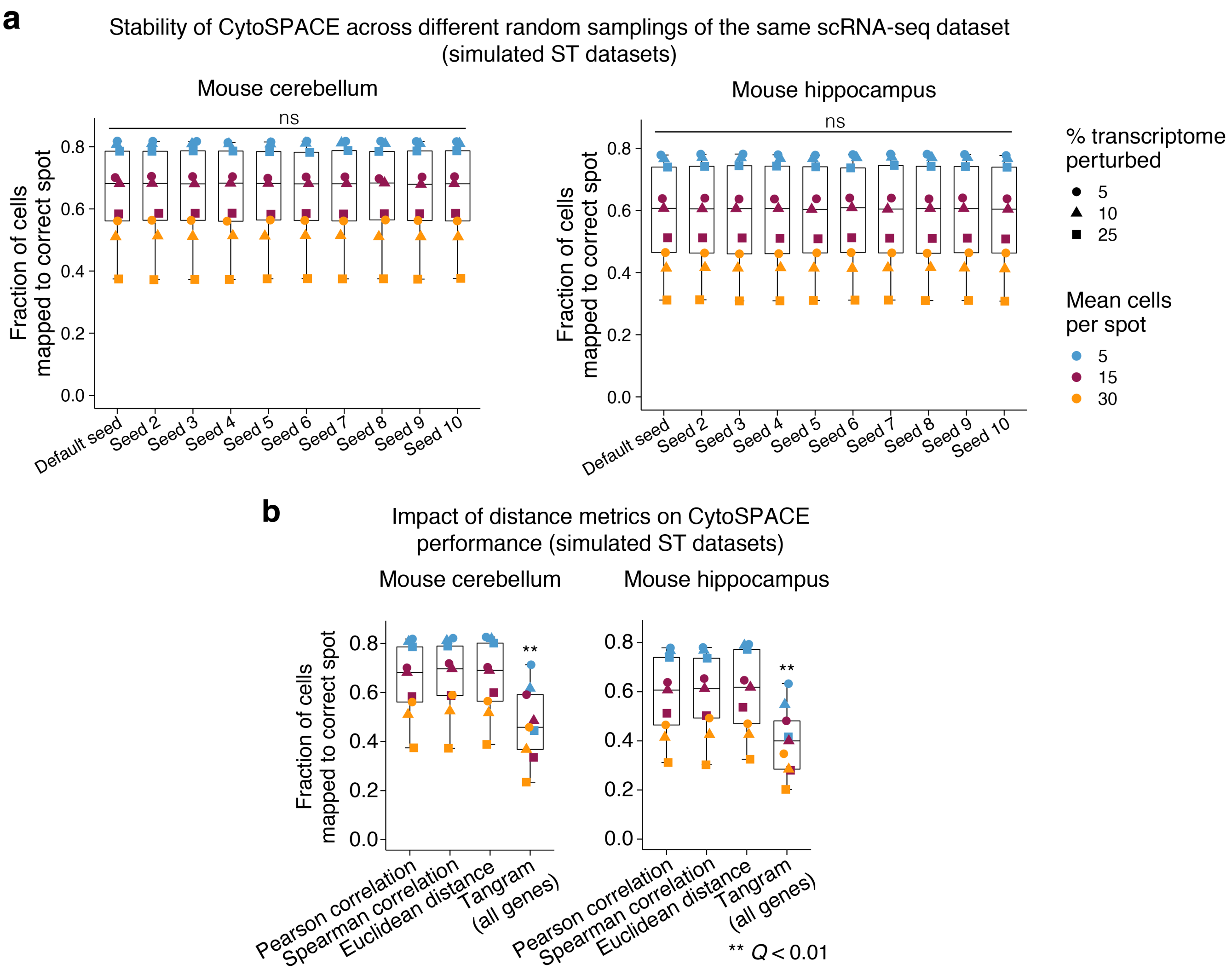

Supplementary Figure 4: Stability of CytoSPACE cell-to-spot assignments across multiple seeds and distance metrics. a, Same as **Extended Data Figure 4** but showing CytoSPACE performance for 10 different random samplings of each scRNA-seq query dataset. Statistical significance was calculated using a one-way repeated measures ANOVA. ns, not significant. **b,** Same as **Extended Data Figure 4** but showing CytoSPACE performance on simulated ST datasets using Pearson correlation, Spearman correlation, or Euclidean distance to calculate the CytoSPACE cost matrix. The legend is identical to panel a. Group comparisons were performed using a two-sided paired Wilcoxon test for each CytoSPACE distance metric versus each method in **Extended Data Figure 4**, with “Tangram (all genes)” shown as a representative example. P-values were corrected using the Benjamini-Hochberg method and are expressed as q-values (***Q* < 0.01). Q-values are inclusive of all pairwise comparisons between CytoSPACE and benchmarked methods in **Extended Data Figure 4**.

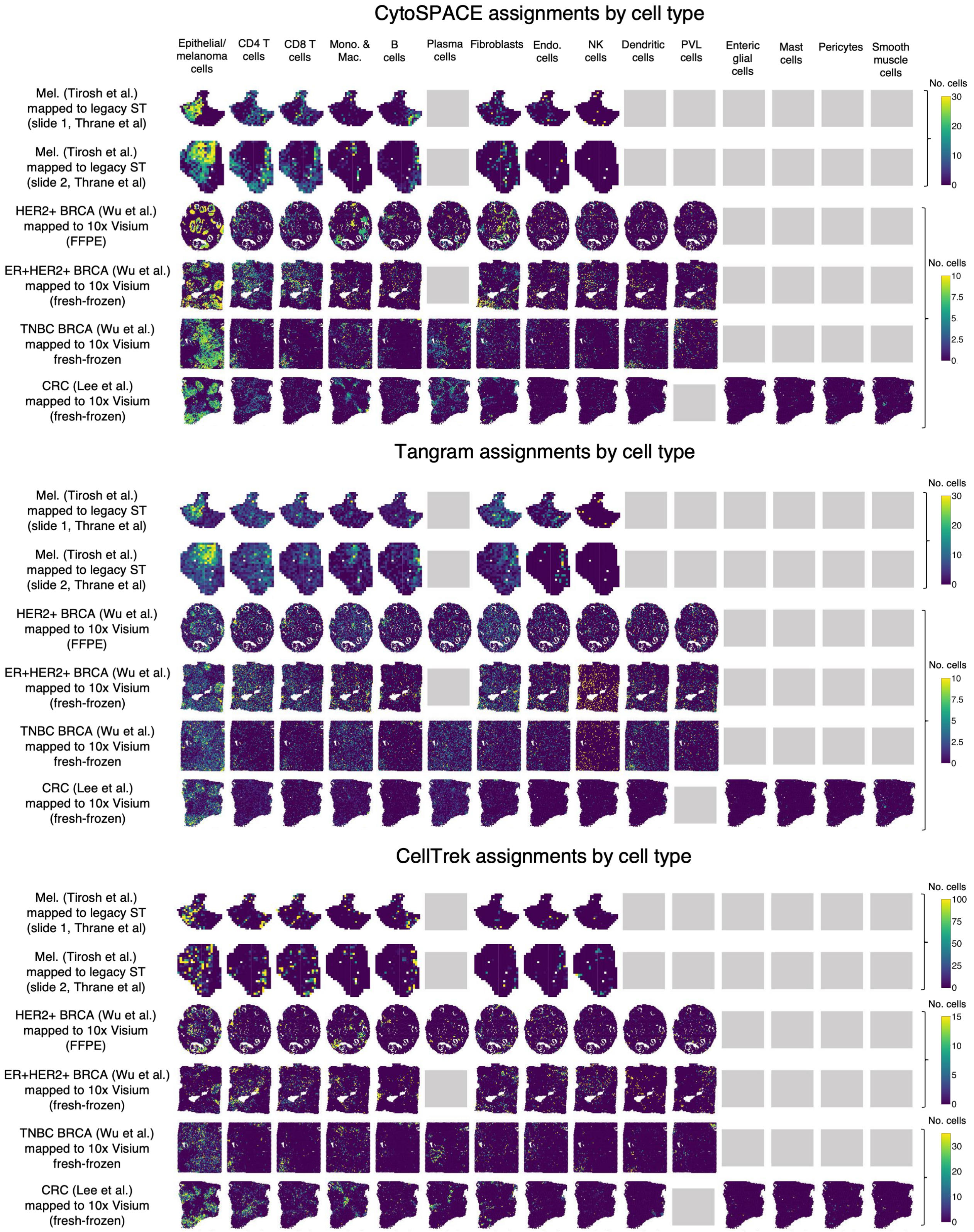

Supplementary Figure 5: Single-cell RNA-seq data mapped onto ST profiles of diverse human tumor specimens. Same as Figure 2a but showing all cell types analyzed for each scRNA-seq/ST dataset by CytoSPACE, Tangram, and CellTrek. Gray boxes denote cell types without author-supplied annotations in the corresponding scRNA-seq atlas (**Methods**).

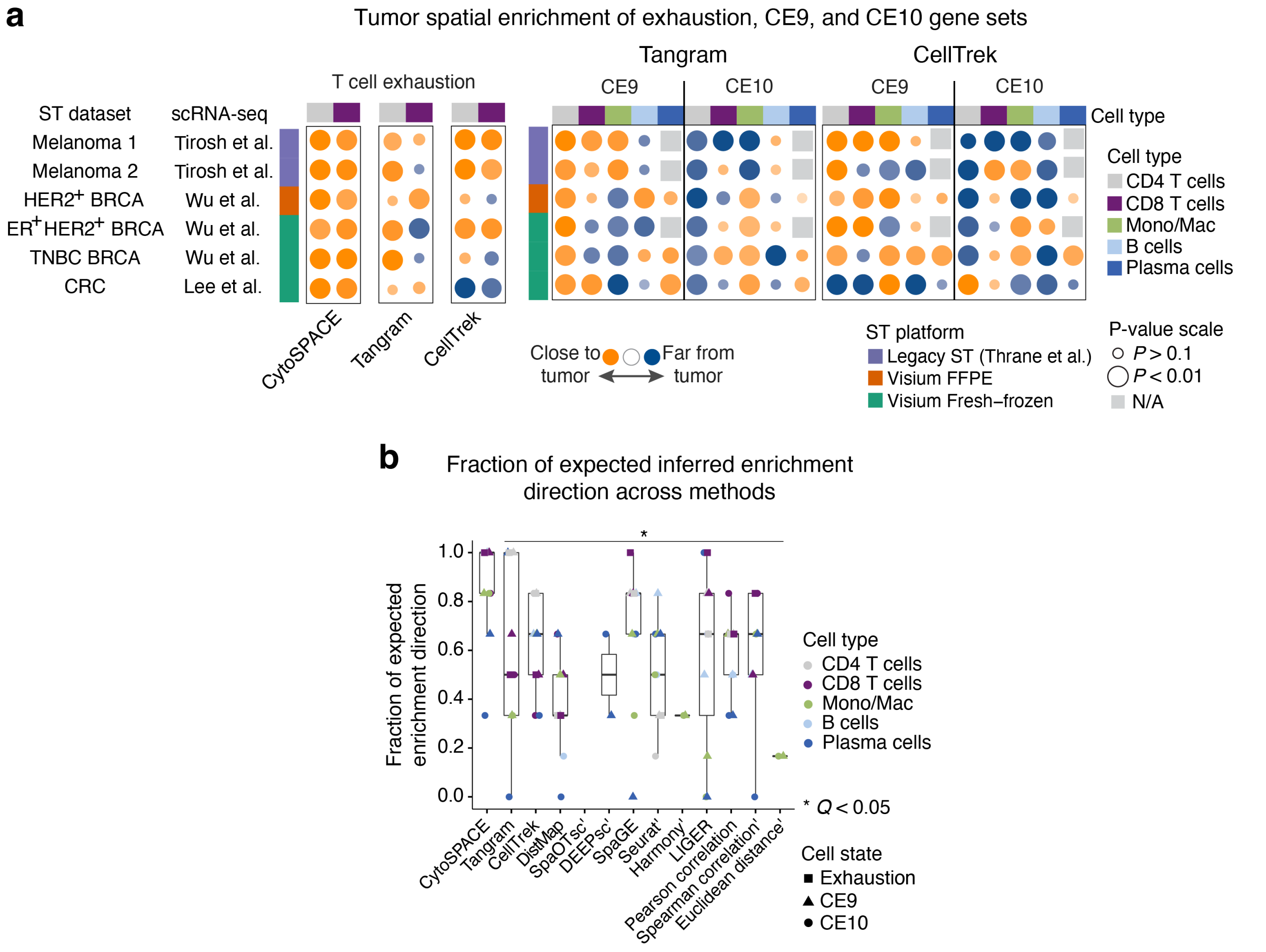

Supplementary Figure 6: Spatial enrichment of tumor-associated cell states across methods and datasets. a, *Left:* Bubble plot showing the spatial enrichment of exhaustion genes in CD4 and CD8 T cell transcriptomes mapped onto ST spots by CytoSPACE, Tangram, and CellTrek (related to **Fig. 2d**). *Right:* Same as **Figure 2e** but showing performance for Tangram and CellTrek. Single-cell RNA-seq datasets without annotated plasma cells are indicated by gray boxes (“N/A”). Bubbles denote normalized enrichment scores calculated by pre-ranked GSEA. **b**, Fraction of datasets per cell type for which the expected spatial enrichment direction was correctly inferred by each of the 13 evaluable methods for each of the gene sets analyzed in this work (n = 11 distinct gene sets with 12 data points per method, as canonical exhaustion genes were analyzed for CD4 and CD8 T cells). The box center lines, box bounds, and whiskers indicate the medians, first and third quartiles and minimum and maximum values within 1.5× the interquartile range of the box limits, respectively. Statistical significance was determined using a two-sided paired Wilcoxon test relative to CytoSPACE, with p-values corrected using the Benjamini-Hochberg method. Methods indicated by a prime symbol failed to map all evaluated cell types (for further details, see the caption of **Fig. 2f**).

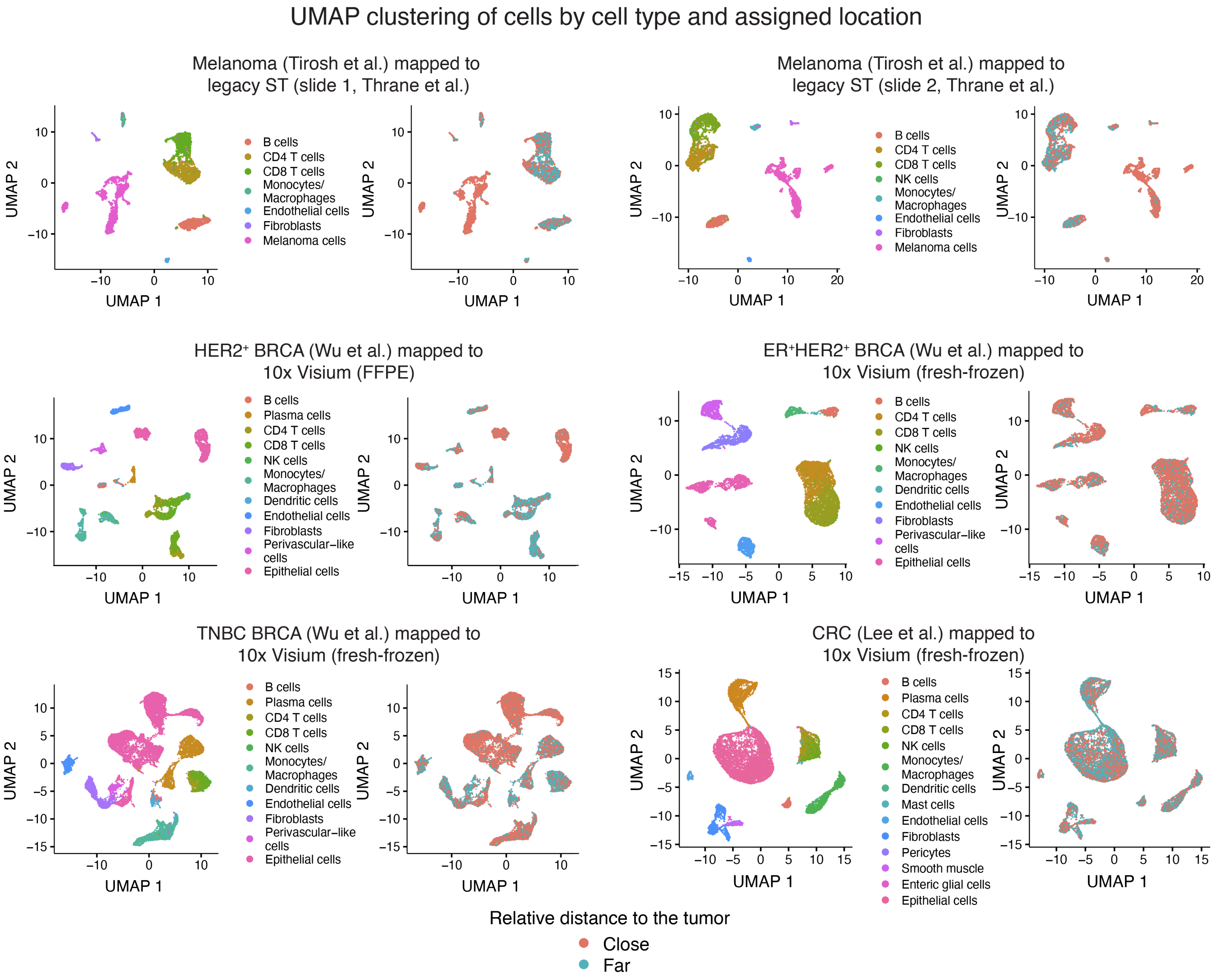

Supplementary Figure 7: UMAP projections of scRNA-seq tumor atlases labeled by predicted spatial locations. UMAP embeddings showing all single-cell transcriptomes mapped by CytoSPACE to ST samples analyzed in Figure 2. Cells are colored by lineage (left) and by relative distance to tumor cells (right), determined as described in **Methods**.

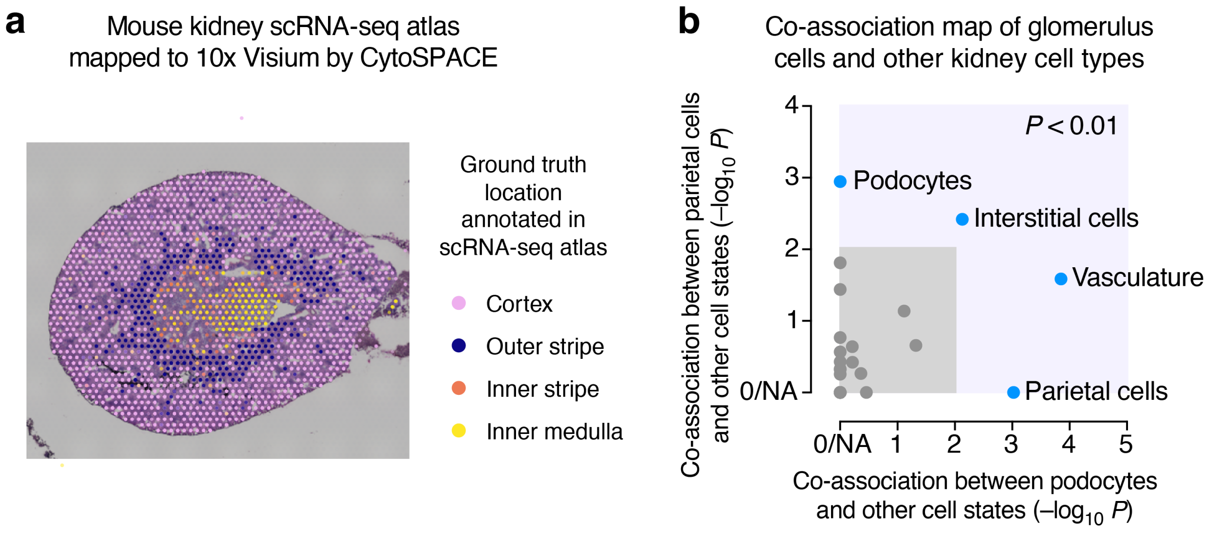

Supplementary Figure 8: Single-cell spatial cartography of the normal mouse kidney using CytoSPACE. a, Mouse kidney scRNA-seq atlas[1] mapped onto a 10x Visium sample of normal mouse kidney[2], shown for epithelial cell transcriptomes mapped by CytoSPACE and colored by the known zonal region of each cell (as in **Fig. 2g**) superimposed over the Visium histological image. Zone colors of individual epithelial cells mapped by CytoSPACE are averaged per spot (for single-cell view, see **Fig. 2h**, top). **b**, Scatter plot showing the statistical significance of co-association between podocytes (epithelial state 1) and all other cell types/states mapped by CytoSPACE (x-axis), and the same for parietal cells (epithelial state 2). Spots were scored as 'present' if at least one cell of a given cell type was mapped by CytoSPACE, and 'absent' otherwise. Significance of co-association was subsequently calculated using a two-sided Fisher's exact test and represented as $-\log_{10}$ p-values. Self-comparisons are denoted by NA (not applicable).

Supplementary References

1. Ransick, A., *et al.* Single-Cell Profiling Reveals Sex, Lineage, and Regional Diversity in the Mouse Kidney. *Developmental Cell* **51**, 399-413.e397 (2019).
2. Ferreira, R.M., *et al.* Integration of spatial and single-cell transcriptomics localizes epithelial cell–immune cross-talk in kidney injury. *JCI Insight* **6**(2021).
