# Supplementary material for: High-resolution alignment of single-cell and spatial transcriptomes with CytoSPACE
Source: Nat Biotechnol. 2023 Mar 6;41(11):1543–8. doi: 10.1038/s41587-023-01697-9 (PMC10635828; doi:10.1038/s41587-023-01697-9)
Supplement: Supplementary file 2 — Reporting Summary [file 41587_2023_1697_MOESM2_ESM.pdf]

nature portfolio

Corresponding author(s): Aaron M. Newman

Last updated by author(s): Jan 18, 2023

Reporting Summary

Nature Portfolio wishes to improve the reproducibility of the work that we publish. This form provides structure for consistency and transparency in reporting. For further information on Nature Portfolio policies, see our Editorial Policies and the Editorial Policy Checklist.

Statistics

For all statistical analyses, confirm that the following items are present in the figure legend, table legend, main text, or Methods section.

n/a	Confirmed	
☐	☒	The exact sample size (*n*) for each experimental group/condition, given as a discrete number and unit of measurement
☐	☒	A statement on whether measurements were taken from distinct samples or whether the same sample was measured repeatedly
☐	☒	The statistical test(s) used AND whether they are one- or two-sided<br>*Only common tests should be described solely by name; describe more complex techniques in the Methods section.*
☐	☒	A description of all covariates tested
☐	☒	A description of any assumptions or corrections, such as tests of normality and adjustment for multiple comparisons
☐	☒	A full description of the statistical parameters including central tendency (e.g. means) or other basic estimates (e.g. regression coefficient) AND variation (e.g. standard deviation) or associated estimates of uncertainty (e.g. confidence intervals)
☐	☒	For null hypothesis testing, the test statistic (e.g. *F*, *t*, *r*) with confidence intervals, effect sizes, degrees of freedom and *P* value noted<br>*Give P values as exact values whenever suitable.*
☒	☐	For Bayesian analysis, information on the choice of priors and Markov chain Monte Carlo settings
☒	☐	For hierarchical and complex designs, identification of the appropriate level for tests and full reporting of outcomes
☐	☒	Estimates of effect sizes (e.g. Cohen's *d*, Pearson's *r*), indicating how they were calculated

Our web collection on statistics for biologists contains articles on many of the points above.

Software and code

Policy information about availability of computer code

Data collection	No specific software was used for data collection as data were collected solely from preexisting publicly available data sets.
Data analysis	CytoSPACE v1.0 was coded in Python v3.8 and used to generate the results in this work. It is freely available, along with documentation, vignettes, and helper R scripts for creating CytoSPACE inputs and for estimating cell type fractions with Seurat, at https://github.com/digitalcytometry/cytospace. CytoSPACE package dependencies include numpy, lap, scipy, pandas, matplotlib, ortools, and lapjv. The specific versions used for this study are v1.22.2, v0.4.0, v1.8.0, v1.4.1, v3.5.1, v9.2.9972, and v1.3.14, respectively. Additional software packages used for analyses in this study are detailed in Methods and include Python v3.8, R v3.5.1 and 4.0.2+, MATLAB_R2019a, Prism 9+ (Graphpad Software, La Jolla, CA), Seurat (v3.2.3, v4.0.0, v4.0.1, v4.1.0, v4.1.1), fgsea v1.14.0 and v1.20.0, RCTD (v2.0.0, R package spacexr), igraph v1.2.6, and e1071 v1.7.8. Comparative analyses against other methods were performed with the following versions: Tangram v1.0.2, CellTrek v0.0.0.9000, DistMap v0.1.1, SpaOTsc v0.2, DEEPsc (version number not available; last GitHub commit when cloned: June 5, 2022), SpaGE (version number not available; last GitHub commit when cloned: July 20, 2021), Spatial Seurat v3.2.3, Harmony v0.1, and LIGER v1.0.0.

For manuscripts utilizing custom algorithms or software that are central to the research but not yet described in published literature, software must be made available to editors and reviewers. We strongly encourage code deposition in a community repository (e.g. GitHub). See the Nature Portfolio guidelines for submitting code & software for further information.

Data

Policy information about availability of data

All manuscripts must include a data availability statement. This statement should provide the following information, where applicable:

- Accession codes, unique identifiers, or web links for publicly available datasets
- A description of any restrictions on data availability
- For clinical datasets or third party data, please ensure that the statement adheres to our policy

All scRNA-seq/ST datasets of real tissue specimens are publicly available, with details provided in the data availability statement and Supplementary Table 1.

Human research participants

Policy information about studies involving human research participants and Sex and Gender in Research.

Reporting on sex and gender	*Use the terms sex (biological attribute) and gender (shaped by social and cultural circumstances) carefully in order to avoid confusing both terms. Indicate if findings apply to only one sex or gender; describe whether sex and gender were considered in study design whether sex and/or gender was determined based on self-reporting or assigned and methods used. Provide in the source data disaggregated sex and gender data where this information has been collected, and consent has been obtained for sharing of individual-level data; provide overall numbers in this Reporting Summary. Please state if this information has not been collected. Report sex- and gender-based analyses where performed, justify reasons for lack of sex- and gender-based analysis.*
Population characteristics	*Describe the covariate-relevant population characteristics of the human research participants (e.g. age, genotypic information, past and current diagnosis and treatment categories). If you filled out the behavioural & social sciences study design questions and have nothing to add here, write "See above."*
Recruitment	*Describe how participants were recruited. Outline any potential self-selection bias or other biases that may be present and how these are likely to impact results.*
Ethics oversight	*Identify the organization(s) that approved the study protocol.*

Note that full information on the approval of the study protocol must also be provided in the manuscript.

Field-specific reporting

Please select the one below that is the best fit for your research. If you are not sure, read the appropriate sections before making your selection.

☒ Life sciences ☐ Behavioural & social sciences ☐ Ecological, evolutionary & environmental sciences

For a reference copy of the document with all sections, see nature.com/documents/nr-reporting-summary-flat.pdf

Life sciences study design

All studies must disclose on these points even when the disclosure is negative.

Sample size	No sample-size estimates were performed to ensure adequate power to detect a pre-specified effect size. Suitable group minimums for single cell partitioning into groups for gene set enrichment analysis were imposed as described in Methods. All results were analyzed and interpreted using statistically appropriate techniques as described in Methods.
Data exclusions	Some single cells were excluded using quality control metrics described fully in Methods. From scRNA-seq atlases, exclusions comprised all normal melanocytes; T cells which could not be confidently classified as CD8 or CD4 T cells based on author annotations; and myeloid cells that could not be confidently classified as monocytes/macrophages or dendritic cells based on author annotations. From MERSCOPE data, cells with fewer than 100 transcripts or fewer than 10 genes were excluded from analysis. For gene set enrichment analysis within cell types, cell types which resulted in fewer than 10 cells assigned to a spatial group were excluded from analysis for the corresponding method.
Replication	All attempts at replication were successful, including replication of CytoSPACE performance on both simulated and real datasets across ten independent random seeds as described in Methods.
Randomization	No randomization was applied. Single cells mapped by CytoSPACE or other methods were partitioned deterministically into classes according to the experimental question as described in Methods. Samples were otherwise not divided into groups.
Blinding	No experimental groups were involved in data collection. For data analysis, instances of group allocation consisted of single cells grouped by cell type labels and by spatial regions of interest including tumor/normal boundaries. In all cases, the mapping procedure within CytoSPACE was blinded to cell type labels and spatial regions of interest.

Reporting for specific materials, systems and methods

We require information from authors about some types of materials, experimental systems and methods used in many studies. Here, indicate whether each material, system or method listed is relevant to your study. If you are not sure if a list item applies to your research, read the appropriate section before selecting a response.

Materials & experimental systems

n/a	Involved in the study
☒	☐ Antibodies
☒	☐ Eukaryotic cell lines
☒	☐ Palaeontology and archaeology
☒	☐ Animals and other organisms
☒	☐ Clinical data
☒	☐ Dual use research of concern

Methods

n/a	Involved in the study
☒	☐ ChIP-seq
☒	☐ Flow cytometry
☒	☐ MRI-based neuroimaging
